# Deep sequencing–based comparative transcriptional profiles of *Cymbidium hybridum* roots in response to mycorrhizal and non-mycorrhizal beneficial fungi

**DOI:** 10.1186/1471-2164-15-747

**Published:** 2014-08-31

**Authors:** Xiaolan Zhao, Jianxia Zhang, Chunli Chen, Jingze Yang, Haiyan Zhu, Min Liu, Fubing Lv

**Affiliations:** Guangdong Key Laboratory for Innovative Development and Utilization of Forest Plant Germplasm, South China Agricultural University, Wushan Road 483, Tianhe District, Guangzhou 510642 People’s Republic of China; Key Laboratory of South China Agricultural Plant Genetics and Breeding, South China Botanical Garden, The Chinese Academy of Sciences, Guangzhou, 510650 People’s Republic of China; Guangdong Key Laboratory of Ornamental Plant Germplasm Innovation and Utilization, Environmental Horticulture Research Institute, Guangdong Academy of Agricultural Sciences, East 1st Street 1, Jinying Road, Tianhe District, Guangzhou 510640 People’s Republic of China

**Keywords:** Root transcriptome, Digital gene expression, Plant mycorrhizal symbiosis, *Cymbidium hybridum*, *Epulorhiza repens* ML01, *Umbelopsis nana* ZH3A-3

## Abstract

**Background:**

The Orchidaceae is one of the largest families in the plant kingdom and orchid mycorrhizae (OM) are indispensable in the life cycle of all orchids under natural conditions. In spite of this, little is known concerning the mechanisms underlying orchid- mycorrhizal fungi interactions. Our previous work demonstrated that the non-mycorrhizal fungus *Umbelopsis nana* ZH3A-3 could improve the symbiotic effects of orchid mycorrhizal fungus *Epulorhiza repens* ML01 by co-cultivation with *Cymbidium hybridum* plantlets. Thus, we investigated the *C. hybridum* transcript profile associated with different beneficial fungi.

**Results:**

More than 54,993,972 clean reads were obtained from un-normalized cDNA library prepared from fungal- and mock- treated *Cymbidium* roots at four time points using RNA-seq technology. These reads were assembled into 16,798 unique transcripts, with a mean length of 1127 bp. A total of 10,971 (65.31%) sequences were annotated based on BLASTX results and over ninety percent of which were assigned to plant origin. The digital gene expression profiles in *Cymbidium* root at 15 days post inoculation revealed that 1674, 845 and 1743 genes were sigificantly regulated in response to ML01, ZH3A-3 and ML01+ ZH3A-3 treatments, respectively. Twenty-six genes in different regulation patterns were validated using quantitative RT-PCR. Our analysis showed that general defense responses were co- induced by three treatments, including cell wall modification, reactive oxygen species detoxification, secondary biosynthesis and hormone balance. Genes involved in phosphate transport and root morphogenesis were also detected to be up-regulated collectively. Among the OM specifically induced transcripts, genes related to signaling, protein metabolism and processing, defense, transport and auxin response were identifed. Aside from these orchid transcripts, some putative fungal genes were also identified in symbiotic roots related to plant cell wall degradation, remodeling the fungal cell wall and nutrient transport.

**Conclusion:**

The orchid root transcriptome will facilitate our understanding of orchid - associated biological mechanism. The comparative expression profiling revealed that the transcriptional reprogramming by OM symbiosis generally overlapped that of arbuscular mycorrhizas and ectomycorrhizas. The molecular basis of OM formation and function will improve our knowledge of plant- mycorrhzial fungi interactions, and their effects on plant and fungal growth, development and differentiation.

**Electronic supplementary material:**

The online version of this article (doi:10.1186/1471-2164-15-747) contains supplementary material, which is available to authorized users.

## Background

All orchids produce minute, endosperm- lacking seeds and are dependent on fungal colonization for germination and growth into an underground heterotrophic, achlorophyllous stage called a protocorm under natural conditions [1, 2]. After this fully mycotrophic stage, most species shift to autotrophy at adult stage with the development of photosynthetic organs, but approximately 200 species worldwide stay fully mycoheterotrophic (MH) [3]. Recently, some chlorophyllous species in Orchidaceae were verified to obtain carbon at adult stage not only from their photosynthetic activity, but also from mycorrhizal fungi, which were called mixotrophs (MX) [4, 5]. Among these, several terrestrial orchids such as *C. lancifolium* and *C. goeringii* were shown to be MX nutritional mode through isotopic analyses [6]. The dependency of green orchids on mycorrhizal fungi throughout their life cycle prompts us to study these mycorrhizal interactions in more detail.

In nature, orchids largely associate with members of the ‘rhizoctonia’ complex, which contains three now taxonomically disparate Agaricomycetes (¼Hymenomycetes) taxa: Sebacinales, Ceratobasidiaceae and Tulasnellaceae. Tulasnellaceae are the most frequently found ‘rhizoctonias’ in green orchids [1, 3]. Besides orchid mycorrhizal fungi (OMF), recent studies also demonstrated that isolates of *Umbelopsis*, as non-mycorrhizal beneficial endophytes, probably co-existed in the roots of wild orchids [7, 8].

*C. hybridum* collectively contains a large number of cultivars with large flowers. Over 20 species in *Cymbidium*, including *C. sinense, C. goeringii*, *C. faberi, C. ensifolium* and *C. kanran*, were used in their breeding programs. In our previous work, one OMF isolate ML01 and one non-mycorrhizal beneficial isolate ZH3A-3 were isolated from roots of wild *C. spp.* adult plants and identified as *E. repens* [ITS accession number of ML01 in GenBank: KJ499806; also referred to *Tulasnella*-like *Rhizoctonia*] and *U. nana* [ITS accession number in GenBank: HM214447], respectively. Here, we present that ML01 forms typical mycorrhiza with *C. hybridum*. Individual (ML01, ZH3A-3) or combinative (ML01 + ZH3A-3, MZ) inoculation significantly improved the vegetative growth of *C. hybridum* plantlets compared with mock-inoculated controls.

Although orchid mycorrhizal (OM) symbiosis is an important feature in the life cycle of orchids , it has long been considered to be an atypical mycorrhizal association, with the fungus deriving little benefit from the orchid host [3]. But Cameron *et al.* [9, 10] reported that approximately 0.4–3.0% of the labeled carbon provided to the orchids was passed to the fungal partner [9, 10]. Thus adult orchid mycorrhizas may represent a truly mutualistic interaction similar to ectomycorrhizae (ECMs) and arbuscular mycorrhizae (AMs) associations [3].

Compared with the numerous transcriptional analyses to identify genes involved in AM [11–15] and ECM symbiosis [15–17] , the molecular mechanisms behind OM symbiosis are still poorly understood [18–21]. Recently, different gene expression or proteome changes of orchid protocorms in response to mycorrhizal fungi were studied and mutualistic plant-fungus relationship was suggested [19–21]. As for orchids at the adult stage, Valadares [20] used RNA-seq to determine the gene expression profile of wild *Oeceoclades maculata* mycorrhizal roots in comparison to non-mycorrhizal controls [22]. While this study provides new insights into our understanding of the OM symbiosis for adult orchids, the symbiosis seems to be highly complex as wild orchids generally associate with multiple symbionts even within a single root [23]. Although genomic sequence resources currently available for orchids are limited, large expressed transcripts from *Phalaenopsis aphrodite*, *P. equestris*, *P. bellina*, *Oncidium* Gower Ramsey, *Erycina pusilla* and *C. sinense* were generated in recent years with the rapid advances in DNA sequencing technology known as next-generation sequencing (NGS or massively parallel sequencing) and the development of bioinformatic processes, especially for de novo assembly without reference genome information [24–26]. Whereas no genetic information on orchid mycorrhizal fungi was available when our analyses began, large numbers of assembled transcripts and the genome information of *T. calospora* had been recently generated as part of a DOE JGI Community Sequencing program. This work has allowed us to assign some putative fungal genes to mycorrhizal fungi ML01 by Blastn searches of these data. In this paper, we used NGS approach and Illumina Digital Gene Expression (DGE) technology to obtain genome-wide insight into the transcriptomic responses of *C. hybridum* under specific interactions with mycorrhizal or/and other beneficial fungi. To our knowledge, this represents the first genome-wide gene expression profile of orchid plants in response to mycorrhizal or non-mycorrhizal fungi. The data enabled us to get new insight into the mechanisms underlying plant responses to mycorrhizal and non-mycorrhizal beneficial fungi. The transcriptome for *C. hybridum* could also be used as an important resource for exploring other biological processes in orchids.

## Methods

### Biological materials and inoculation

The micro-propagated plantlets of *C. hybridum* ‘Golden Boy’ were provided by the Guangdong Key Lab of Ornamental Plant Germplasm Innovation and Utilization, China. The propagation procedure for orchid plantlets was described in [8]. The two fungal isolates ML01 and ZH3A-3 were preserved in the Guangdong Key Laboratory for Innovative Development and Utilization of Forest Plant Germplasm. Inocula were produced by growing each strain on 90 mm Petri dishes containing potato-dextrose agar (PDA) for 10 days.

The plantlets with 3–4 roots about 2.5-3 cm long were then transferred to DE basal medium, an *in vitro* mycorrhizal induction medium in orchids [27]. The co-cultivation DE medium contained 1.0 mM CaCl_2_, 0.5 mM MgS0_4_, 1.0 mM K_2_S0_4_, 0.4 mM KH_2_P0_4_, 100 μM FeS0_4_, 25 μM H_3_BO_4_, 33 μM MnCl_2_, 2.8 μM ZnSO_4_, 1.0 μM NaMoO_4_, 140 μM Na_2_EDTA, supplemented with yeast extract 1 g · L^-1^, soluble starch 9 g · L^-1^ and agar 6 g · L^-1^. Each plantlet was inoculated with a 7 mm plug excised from an edge of an actively growing colony on PDA of strain ML01, ZH3A-3 or ML01+ ZH3A-3.The control plantlets were mock-inoculated with a plug excised from a PDA plate without fungus. All the treated and control plantlets were then incubated under a photoperiod of 16 hrs light at a temperature of 22°C.

### Symbiotic effects on vegetative growth of *C. hybridum*

After 45 days post- inoculation (dpi), ninety plantlets of each treatment were totally harvested and randomly divided into three groups, which represented three biological replicates. The symbiotic effects on the vegetative growth of the hosts were assessed by total increased fresh weight and dry biomass of the plantlets. The percentage of root growth (PRG) and mycorrhizal dependency (MD) were also compared among different treatments. MD was calculated by the following formula: (shoot dry wt. of mycorrhizal plants– shoot dry wt. of non-mycorrhizal plants) × 100/shoot dry wt. of mycorrhizal plants as described in [28]. For statistical analysis of data, SPSS v13.0 was employed for one-way analysis of variance (ANOVA) and comparisons among means were made using Duncan’s multiple range test, calculated at p < 0.05.

### Root staining and histology

The successful inoculation with strains ML01 and ZH3A-3 and the absence of fungal contaminants in mock-treated roots were confirmed by trypan blue staining [8]. To confirm their respective colonization pattern, semi-thin sections of root samples for each treatment were prepared and stained with a 0.05% aqueous solution of toluidine blue for light microscopy (Olympus-BX51, Olympus Corp., Tokyo, Japan).

### Reference cDNA library construction

At 1, 3, 15 and 30 dpi, orchid roots of MZ- treated or mock-inoculated control plantlets were harvested by carefully removing the substrate and mycelia, and the samples were frozen in liquid nitrogen and stored at -80°C. Total RNA from each sample was extracted separately with Column Plant Total RNAout kit according to the manufacturer’s protocol (TIANDZ Co., Beijing, China). The RNA was treated with DNase (Takara, China) and then quantified using a NanoDrop 2000™ spectrophotometer (Thermo Scientific, Waltham, MA, USA). Equal amounts of total RNA from MZ- treated and mock-inoculated samples at each time point were pooled to produce minimum 20 μg total RNA for constructing a cDNA library for establishing the reference transcriptome.

### mRNA-sequencing and cDNA *de novo*assembly

The library construction and sequencing were performed by the Beijing Genomics Institute (BGI) (Shenzhen, China) and the general experimental pipeline was summarized by previously published papers [29, 30]. First, the cDNA library was pair-end sequenced by Illumina HiSeq2000 (90 bp single read module). After removing adaptor sequences, empty reads and low quality sequences (reads with unknown sequences ‘N'), we obtained the clean reads for *de novo* assembly. Three *de novo* assembly software were used including SOAPdenovo with optimized *k*-mer length of 41, ABySS [31] with K-mer length of 49 and Trinity [32] (http://trinityrnaseq.sourceforge.net/) with default *k*-mer length of 25. Sequence directions of the resulting unigenes were validated by blastx alignment (e-value < 0.00001) between unigenes and public protein databases with a priority order of NR (non-redundant protein sequences in NCBI), Swiss-Prot, Kyoto Encyclopedia of Genes and Genomes database (KEGG), and COG. When a unigene happens to be unaligned to none of the above databases, ESTScan will be introduced to predict its coding regions as well as to decide its sequence direction.

After sequencing and assembling, we obtained three *de novo* assembled transcriptome database with three different assembly methods. The number of assembled transcripts, total bases of transcripts, average length of all unigenes, N50, number of long-transcripts (≥1 kb), and number of reads that could be mapped back to transcripts (RMBT) were compared to evaluate the assembly quality with different methods and select the optimum reference transcriptome for further study. The expression level of unigene was measured by the number of clean reads mapped to each annotated unigene normalized to RPKM (reads per Kb per million reads) [33] and adjusted using an additional normalizing factor [34].

### Functional annotation

The protein functional annotation of unigenes was given according to the known proteins with the highest sequence similarity by Blastx to public protein databases mentioned above. Domain-based alignments were carried out against the COG database to predict and classify possible functions of unigenes. We further used the Blast2GO program (http://www.blast2go.org) to get GO annotation of unigenes.

### Validation of the reference assembly

To validate the assembled reference transcripts, 18 unigenes were randomly chosen for further RT-PCR and Sanger sequencing. Gene specific primers were designed according to the assembled transcripts with primer premier software (version 5.0) (Additional file 1: Table S1). The reaction was performed in 25 μl, containing 0.5 μl cDNA template, 12.5 μl Ex Taq Mix (TAKARA, Qingdao, China), 1 μl of each 20 μM forward and reverse gene-specific primers and 10 μl of PCR-grade water. Touch-down PCR reactions were performed as follows: 95°C for 5 min before cycling 30 rounds of 94°C for 30 s, 65°C for 30 s (decreasing 0.5°C in every cycle) and 72°C for 1 min 30 s – followed by 15 cycles of 94°C for 30 s, 54°C for 30 s and 72°C for 1 min 30 s and finally a 72°C extension step for 10 min. The specificity of products was inspected by 1% agarose gel electrophoresis before they were purified with Ezgene™ Gel/PCR Extraction Kit (DC3511-02, Biomiga). The purified PCR products were Sanger-sequenced by the Beijing Genomics Institute (BGI) (Shenzhen, China).

### Identification of differentially expressed genes (DEGs) using Illumina short reads

To perform quantitative comparisons of the transcript variation with respect to different beneficial fungi, total RNA samples generated from roots treated with ML01 (CyEX21), ZH3A-3 (CyEX22) or ML01 + ZH3A-3 (CyEX23 ) and mock-inoculated controls (CyEX20) at 15 dpi were used to construct un-normalised cDNA libraries which were sequenced by Illumina HiSeq2000 (100 bp single read module). The cDNA library construction, sequencing, transcript profiling and mapping back to the newly assembled reference transcriptome with Trinity were carried out as a custom service (Beijing Novogene Bioinformatics Technology Co., Ltd., Beijing, China). The expression level of each assembled transcript in different samples was measured as RPKM values. SeqMap [35] was used for read mapping and rSeq [36] was applied for RPKM based expression measurement. Abundance data from different samples were collected for each transcript. In our work, the changes in relative abundance for unigenes between two samples were screened with the threshold: q-value ≤ 0.005 and log2Ratio ≥ 1 or ≤ -1.

### Quantitative RT-PCR (qRT-PCR) validation

qRT-PCR was further carried out to analyze the expression of twenty- six DEGs identified by RNA-seq technology. Gene specific primers were designed according to the cDNAs with Primer Premier software (version 5.0) (Additional file 2: Table S2). For RNA extraction of each treatment, three biological replicates were collected independently and immediately frozen in liquid nitrogen. One microgram of total RNA was reverse-transcribed in a 20 μL reaction mixture from a PrimeScript II 1st Strand cDNA Synthesis Kit (Takara, Dalian, China). qRT-PCR was performed in a 25 μL reaction mixture containing 2 × SYBR Master Premix Ex Taq II 12.5 μL (Takara, Dalian, China), 1 μL of cDNA template (1:5 dilution), and 1 μL of each corresponding primer for the gene of interest. qRT-PCR of three biological replicates for each sample was performed using a LightCycler 480 II System with its relative quantification software (ver. 1.2) based on the delta-delta-Ct method (Roche). qRT-PCR was performed for 5 s at 95°C, 10 s at 56°C, and 20 s at 72°C. The cDNA samples were standardized to two reference genes: B21 (Unigene11515-all) and B24 (Unigene1346-all).

## Results

### Co-cultivation of *C. hybridum*plantlets with different fungi and their respective symbiotic effects on host vegetative growth

When co-cultivated with *C. hybridum* plantlets, the hyphae of the two isolates ML01 and ZH3A-3 began to grow at 2 dpi and first contacted the hosts’ roots at 4 dpi. After 15 dpi, the co-cultivation media of plantlets inoculated with ML01 changed from transparent to opaque, whereas the actively growing white fluffy hyphae of isolate ZH3A-3 fully covered the plate. The hyphal growth of isolate ZH3A-3 was retarded by the co-inoculated isolate ML01, which showed space at the colony margin (Additional file 3: Figure S1). After 45 dpi, ML01, ZH3A-3 and MZ treatments all significantly improved the total fresh weight and dry biomass of *C. hybridum* plantlets compared to the control (Table 1).

**Table 1:** **Effect of inoculation with** ***Epulorhiza repens*** **isolate ML01and/or** ***Umbelopsis nana*** **isolate ZH3A-3 on the growth parameters of** ***Cymbidium hybridum*** **plantlets at 45 dpi**

Treatment	PRG (%)	Leaf FW (g)	Root FW (g)	Increased FW (g)	Leave DW (g)	Total DW (g)	MD (%)
control	22	0.76 ± 0.09^b^	0.35 ± 0.13^d^	0.34 ± 0.11^d^	0.07 ± 0.01^b^	0.09 ± 0.02^c^	0
ML01	53.3	1.19 ± 0.14^a^	0.49 ± 0.20^cd^	0.79 ± 0.17^bc^	0.13 ± 0.04^a^	0.17 ± 0.04^ab^	46.5
ZH3A-3	62.5	1.00 ± 0.12^a^	0.71 ± 0.13^ab^	0.84 ± 0.15^ab^	0.12 ± 0.03^a^	0.17 ± 0.04^ab^	41.7
ML01 + ZH3A-3	79.0	1.11 ± 0.12^a^	0.89 ± 0.21^a^	0.99 ± 0.14^a^	0.14 ± 0.05^a^	0.20 ± 0.07^a^	50.0

All the data were mean of three biological replicates, ± standard deviation. (FW: fresh weight; DW: dry weight; MD (%): percentage of mycorrhizal dependency; PRG (%): the elongated root number/total root number × 100). Values followed by different lower-case letters within a column are significantly different at P < 0.05 according to DMRT.

### Detection the colonization feature of different beneficial fungi on roots of *C. hybridum*plantlets

In an initial observation, the hyphae of two isolates began to contact with orchids roots at 4 dpi. After that time point, we stained the host's roots with each treatment every two days to monitor the colonization process of different fungi and the root staining results of each treatment at 6, 10, 15 and 30 dpi were presented in Additional file 4: Figure S2. At 6 dpi, the first contacted roots were stained to confirm the hyphal penetration by isolates ML01 or ZH3A-3. After 15 dpi, orchid roots were extensively colonized by these fungi. At 30 dpi, the symbiotic roots of orchid plantlets showed uneven staining owing to the rapid growth of roots. To confirm the root staining results and characterize the exact colonization pattern of these beneficial fungi, we further analyzed the microscopic structure of the formed symbionts at 15 dpi.

**Figure 1:**
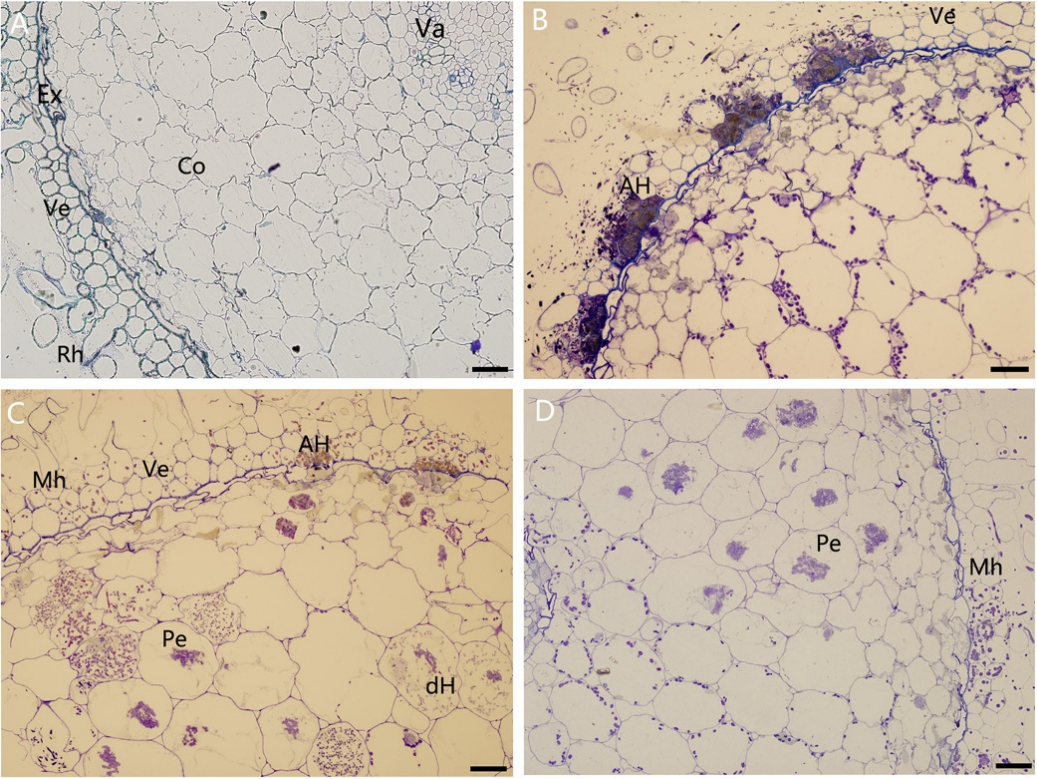
**Microscopic structure of the symbionts formed by different** ***C. hybridum*** **- beneficial fungi interaction at 15 dpi.** Mock-inoculated control **(A)** and inoculated with isolate ZH3A-3 **(B)**, ML01 + ZH3A-3 **(C)** and ML01 **(D)**. AH, aggregated hyphae of isolate ZH3A-3; Co, cortex cells; dH, degenerated hyphae; Ex, exodermis; Mh, fungal hyphae of isolate ML01; Pe, pelotons; Rh, root hair; Va, vascular cylinder; Ve, velamen cells. Bar, 50 μm

Root cross sections of *C. hybridum* plantlets generally included root hairs, velamen (2–3 layers), exodermis (1 layer), cortex (10–13 layers), endodermis (1 layer), pericycle (1 layer), vascular cylinder and pith (Figure 1A). At 15 dpi, typical pelotons were formed by isolate ML01 in the host cortex cells (Figure 1D) whereas hyphae of isolate ZH3A-3 only colonized the velamen cells at regular intervals (Figure 1B). Roots co-inoculated with isolates ML01 and ZH3A-3 contained newly formed and degenerated pelotons, and also typical aggregated hyphae of isolate ZH3A-3, demonstrating the co-infection of two isolates (Figure 1C). None of these fungal structures were observed in control root tissue (Figure 1A).

### Root transcriptome profile of *C. hybridium*generated by mRNA-sequencing (RNA-seq)

Up to date, none of genome sequences for Orchidaceae was available in public database. RNA-seq, a cost-effective and highly efficient next-generation sequencing technology, provides high throughput and is therefore commonly used for de novo transcriptome assemblies in non-model species, including orchid species [26, 37–39]. To analyze the transcript variation in *C. hybridium* colonized by beneficial fungi, pooled total RNA from eight samples including MZ-treated and mock-inoculated plantlets at each time point were used to generate an informative reference transcriptome database. Totally, 4,949,457,480 base pairs raw data were generated using the Illumina HiSeq2000, yielding 54,993,972 clean reads that were 90 bp in length. The raw reads data are available at the NCBI SRA database with the accession number SRA051368.

Three publicly available assemblers as SOAPdenovo, ABySS and Trinity were used to *de novo* assemble short-read RNA-seq data into transcripts. The assembly software tools as SOAPdenovo and ABySS were chosen because of their previous wide application for the similar *de novo* assembly of RNA-seq data sets [31, 38, 39].Trinity method (release 20110519), one of the newest de novo assembly methods, was reported to reconstruct of the majority of full-length transcripts in a sample from RNA-seq reads directly, across a broad range of transcript levels [32]. Recent publications also showed that Trinity had a consistently better performance than the other assemblers as SOAPdenovo, ABySS, trans-ABySS, Oases with single K-mer value, when measured by transcript accuracy, integrity and completeness, and sensitivity to assemble transcripts from low to high expression levels [30]. The outcomes of three assemblers in our study were summarized in Table 2. As shown in Table 2, de novo assembly using SOAPdenovo yielded a total of 133,984 unique transcripts (≥150 bp), the largest number of transcripts. In contrast to the SOAPdenovo and ABySS assembly, Trinity assembled the longest mean transcript length (1127 bp), the largest N50 (1614 bp), the largest number of long-transcripts (≥1 kb, 7,170 unigenes), and the highest percentage of RMBT (40.72%). We therefore elected to use the Trinity assembly to retain maximum information for further transcript analysis and the Trinity assembly is also available in Additional file 5.

**Table 2:** **Summary of** ***de novo*** **sequence assembly**

Software	Length distribution of unigene (nt)						N50 (bp)	Mean (bp)	All unigenes (nt)	Length of all unigenes (nt)
	100-300	300-500	500-1000	1000-1500	1500-2000	> = 2000				
Trinity	1,603	3,401	4,624	2,927	1,811	2,432	1614	1127	16,798	18,923,761
	9.55%	20.24%	27.53%	17.42%	10.78%	14.48%				
SOAPdenovo	87826	28,196	14,169	2,692	734	367	349	329	133,984	44,138,252
	65.55%	21.04%	10.58%	2.01%	0.55%	0.27%				
ABySS	No data	11,303	8,406	1,550	405	152	593	585	21,816	12,784,421
		51.80%	38.50%	7.10%	1.86%	0.70%				

### Annotation

**Figure 2:**
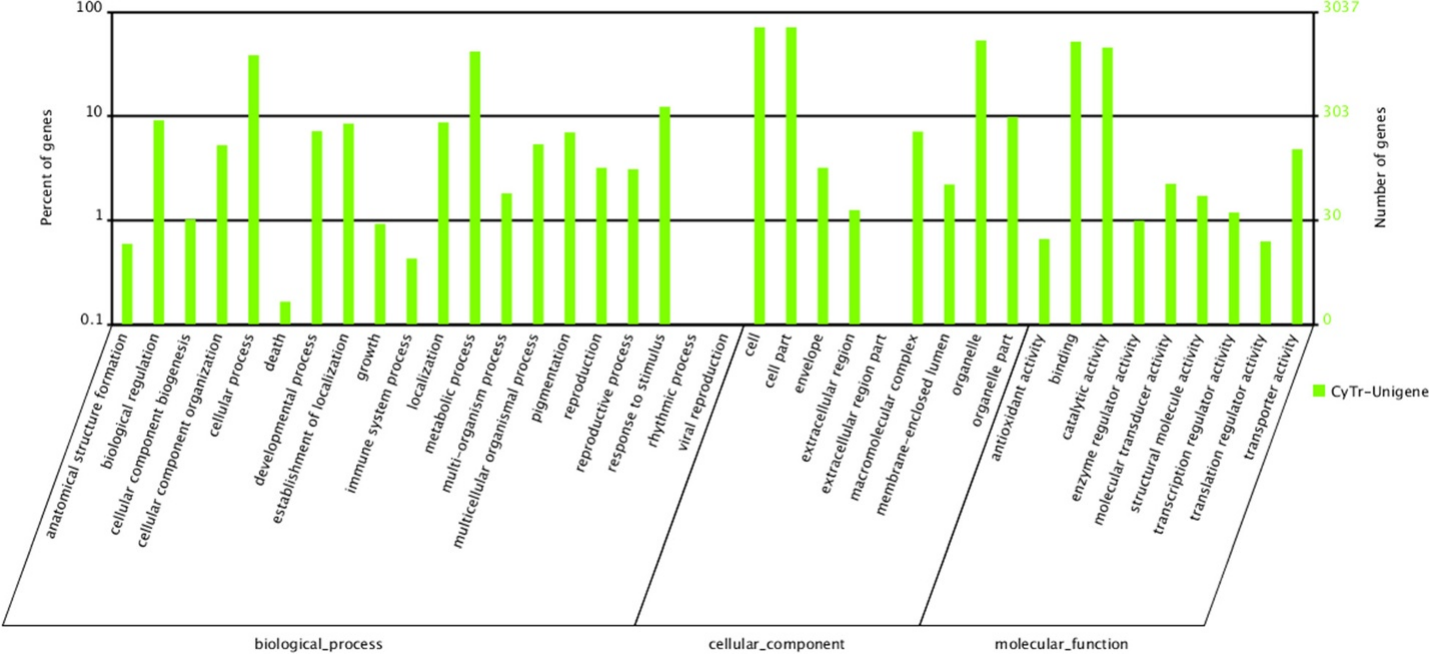
**GO classification of** ***C. hybridum*** **unigenes.**

To validate and annotate the assembled unigenes, the 16,798 unigenes generated by Trinity were subjected to BLASTX searches (E-value ≤ 1e-5) against public protein databases. We found 10,915 (64.98%), 8,486 (50.52%), 5,720 (34.05%) sequences with matches in NR, Swiss-Prot, and KEGG databases, respectively. In total, 10,971 (65.31%) sequences were annotated and another 6,007 (34.69%) unigenes showed no homology to known sequences in these databases. Among the annotated sequences, approximately 690 transcripts shared the highest similarity to fungal or bacterial sequences in NR or Swiss-Prot database. The unigenes homologous to known sequences in public databases were further annotated with GO terms using Blast2GO. A total of 3,037 (18.07%) unigenes were assigned 6,164 GO term annotations in three categories: biological process (27.99%), molecular function (36.32%), and cellular component (35.69%) (Figure 2). Metabolic process, cellular process and response to stimulus rank among the first three categories among the biological processes. The molecular functions most represented were binding, catalytic activity and transporter activity. Cell, cell part and organelle are the most represented categories in cellular components.

**Figure 3:**
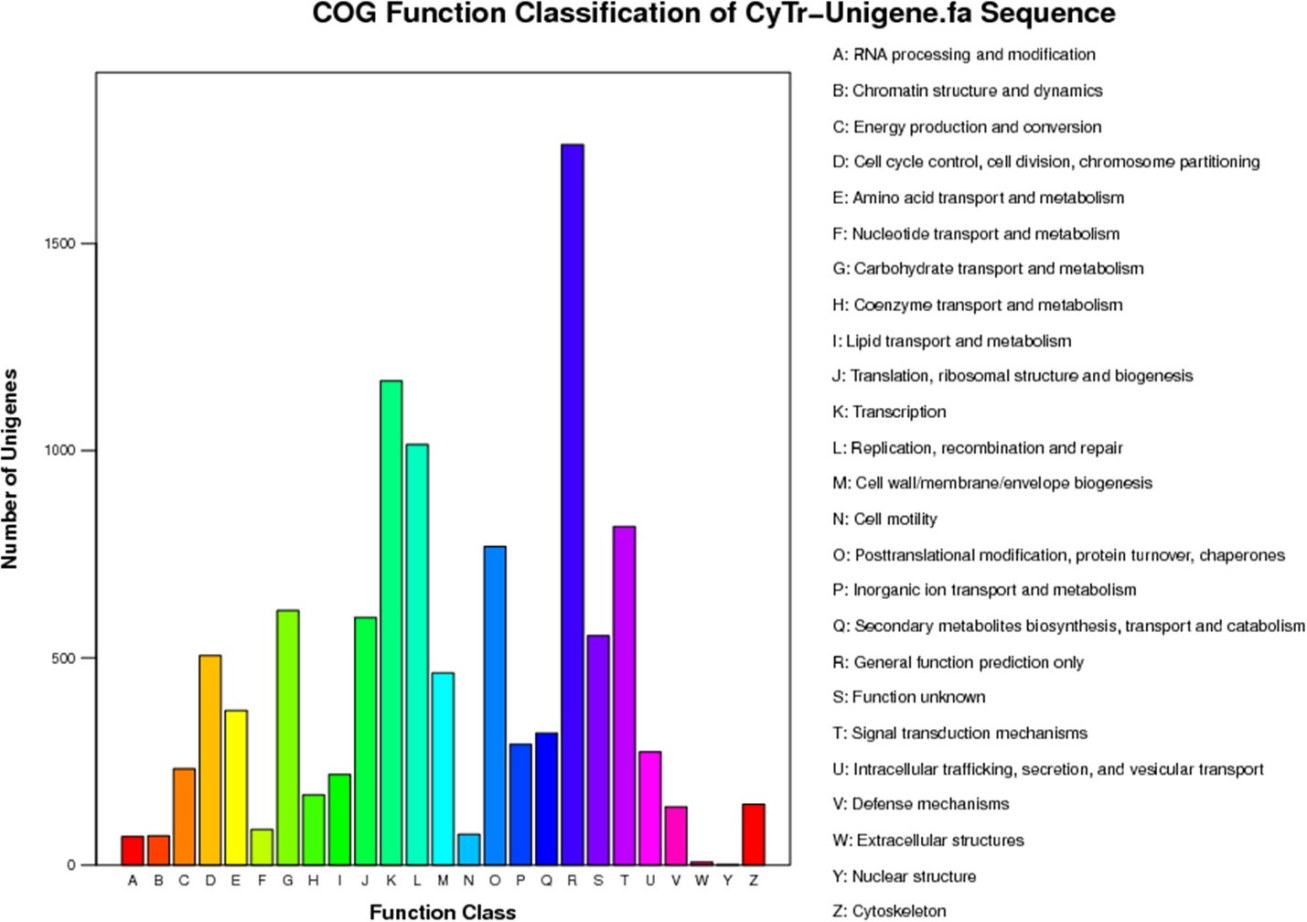
**COG function classification of** ***C. hybridum*** **unigenes.**

The global transcript abundance could be deduced from a non-normalized cDNA library. The transcripts highly abundant in the *Cymbidium* transcriptome are listed in Additional file 6: Table S3. There were 5,721 unigenes mapped into 119 KEGG pathways. Of the 5,721 unigenes, 1,261 (22.04%) were related to metabolic pathways, 545 (9.53%) to biosynthesis of secondary metabolites, 531 (9.28%) to spliceosome, 439 (7.67%) to plant-pathogen interaction, 154 (2.69%) to protein processing in endoplasmic reticulum, and 109 (1.91%) to endocytosis (Additional file 7: Table S4). From the *C. hybridum* transcript database, 4,871of the assembled sequences were assigned to 10,710 COG annotations, which could be grouped into 25 categories (Figure 3). The largest category was “general function prediction only” (16.24%), followed by “transcription” (10.92%), “replication, recombination and repair” (9.48%), “signal transduction mechanisms” (7.63%), “posttranslational modification, protein turnover, chaperones” (7.18%) and “carbohydrate transport and metabolism” (5.73%).

### Validation of assembled transcripts

To experimentally confirm that the unigenes obtained from sequencing and computational analysis were indeed expressed, 18 unigenes were chosen for further RT-PCR and Sanger-sequencing. Blastn results showed that identities of the selected reference assemblies with Sanger-sequencing results were between 96.02-100% (see Additional file 1: Table S1), confirming the reliability of assembled reference transcriptome. These unigenes were also included in the next qRT-PCR analysis.

### Global analysis of differential gene expression in the symbiotic interaction

Having generated a *C. hybridum* root reference transcriptome, our next goal was to perform comparative gene expression profiles in orchid roots inoculated with different beneficial fungi. A total of four independent un-normalised cDNA libraries were sequenced using the Illumina HiSeq2000 100 bp single read module (see Additional file 8: Table S5). For each library, the total number of counts for each read was determined and all the reads were mapped back to the reference transcriptome (see Additional file 9: Table S6). In this study, 10.948 million and 14.859 million reads were uniquely mapped, representing between 57.02% -76.41% of total reads, thus, providing good coverage for the transcript profiles.

**Figure 4:**
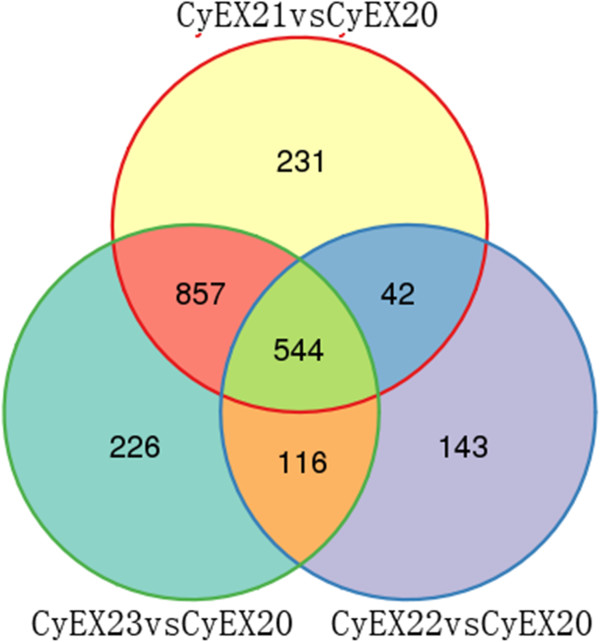
**Venn diagram of differentially expressed genes in the** ***C. hybridum*** **- beneficial fungi interaction.** Mock-inoculated (CyEX20), ML01-treated (CyEX21), ZH3A-3-treated (CyEX22) or ML01 + ZH3A-3 -treated (CyEX23).

The expression level of each assembled transcript sequence in different samples was measured through RPKM values, and the significant changes in the transcript abundance were identified applying a q-value ≤ 0.005 and a two-fold change threshold. Based on these criteria, at 15 dpi, 1674, 845 or1743 genes were significantly regulated in *C. hybridum* root inoculated with isolates ML01, ZH3A-3 or MZ, respectively. The general differential expressed profiles in the three interactions are presented in Figure 4 and Additional file 10: Tables S7-S9. The top 100 abundant genes in symbiotic roots with positive fold change and top 100 abundant genes in non-symbiotic roots with negative fold change in response to symbiotic fungi were presented in Additional file 11: Tables S10 and S11. OM formation specifically induced 57% of the top 100 abundant genes in symbiotic roots with up-regulation pattern. Unigene2357_All (18218.59 RPKM), encoding metallothionein-like protein type 2, which limited oxidative damage through binding to and detoxifying excess copper and other metals, was the most abundant gene in OM symbiotic roots with more than 3.6-fold up-regulation. Other OM-induced genes in this category were largely involved in inhibition of reactive oxygen species (ROS) (glutathione S-transferase and superoxide dismutase [Cu-Zn]), protein metabolism and processing (ribosomal protein, histone H3.2, subtilisin-like protease, serine-type carboxypeptidase, preprotein translocase subunit SecY), signaling (calmodulin-like protein 23, lectin, adhesive/proline-rich protein), cell wall modification (extensin class, beta-mannosidase 4, beta-tubulin) and cell defense and recue (class III chitinase, ATP synthase). Twenty-three genes in this category were co-induced by three treatments, involving in signaling (cytokinin-specific binding protein 1, annexin, calcium-binding EF hand family protein and GTPase), defense (lipoxygenase, GST, class III peroxidase, ubiquitin) and general metabolism (isoflavone reductase, alcohol dehydrogenase, enolase, lipase). However, 63% of the top 100 abundant genes in non-symbiotic roots with negative regulation pattern in response to inoculants were shared by three treatments and another 29% of these genes were specifically down-regulated by OM fungi. The most representative group abundant in non-symbiotic roots and down- regulated by three treatments was attributed to genes encoding main components in photosynthesis system, whereas a mannose-specific lectin, with 49905.50 RPKM in control roots, was specifically down-regulated by OM fungi.

**Figure 5:**
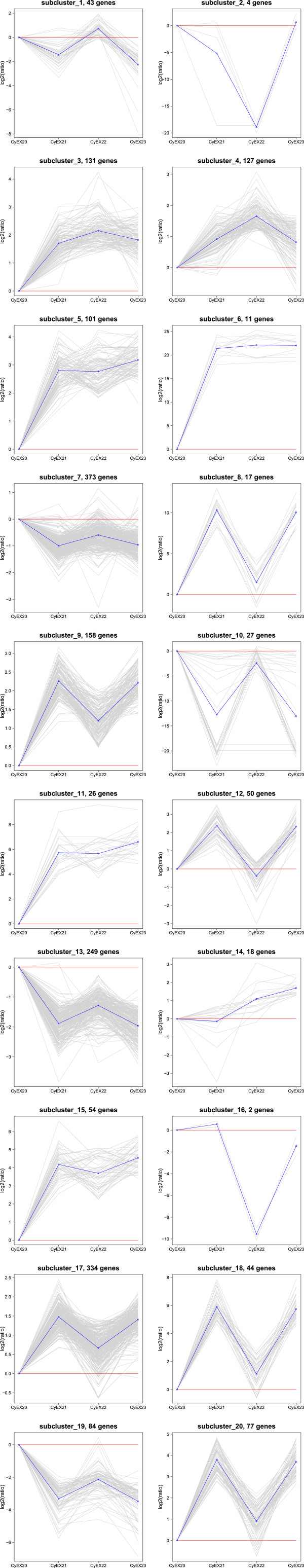
**Twenty regulatory patterns of all the DEGs expressed in different** ***C. hybridum*** **- beneficial fungi interactions based on K-means method.**

As shown in the Venn diagram, 544 DEGS were shared by ML01-, ZH3A-3- and MZ- treatments and 857 transcripts were collectively regulated in CyEX21 and CyEX23 but not in CyEX22 which might specifically functioned in OM mycorrhizal formation. Individual inoculation with non-mycorrhizal fungus ZH3A-3 specifically regulated 143 genes. Another 226 DEGS with special expression pattern in CyEX23 probably functioned in the combinative symbiotic process. All the DEGs were grouped according to hierarchical cluster analysis (Additional file 12: Figure S3) and twenty different expression sub-clusters based on k-means were identified (Figure 5). The lists of all the DEGs in 20 sub-clusters were also presented in Additional file 13. The genes with the same regulation pattern in three treatments were assigned to subcluster 1, 3, 4, 5, 6, 7, 9, 10, 11, 13, 15, 17 and 19. The genes in subcluster 8, 12, 18, 20 were specifically regulated by OM formation. Genes drastically up-regulated by OM fungi were included in subcluster 8, the functions of these genes were related to signaling (serine/threonine protein kinase), reactive oxygen species homeostasis and stress-related (GST, lectin, pathogenesis-related protein 10–3.2), protein metabolism and processing (ribosomal protein L10e, subtilisin, serine carboxypeptidase 1, elongation factor, amino-peptidase) and transport (ABCG, oligopeptide transporter OPT family).

GO annotation was also used to compare the functional distribution of DEGS in different treatments (Additional file 14: Figures S4-S6). Among the represented biological processes, the most represented items were “ion transport” for OM formation and “cellular biosynthetic process” for individual ZH3A-3 treatment. Among the molecular functions, most represented DEGs were categorized as “oxidoreductase activity” for OM formation and “cellulose synthase activity” for individual ZH3A-3 treatment. Among the cellular component, the most represented DEGS were assigned to “extracellular region” for OM formation and “plastid” for individual ZH3A-3 treatment.

To identify the biological pathways regulated during orchid- beneficial fungi interaction, we assigned the DEGs to the reference KEGG pathways (Additional file 15: Tables S12-S14). The top 20 pathways with the most representation by unique sequences for each treatment were included in Figure S7-S9 (see Additional file 16: Figures S7-S9). A large number of unigenes related to primary and secondary metabolism were collectively regulated by three treatments as 'nitrogen metabolism', 'flavonoid biosynthesis', 'cyanoamino acid metabolism', 'pentose phosphate pathway', 'starch and sucrose metabolism', 'alanine, aspartate and glutamate metabolism', 'stilbenoid, diarylheptanoid and gingerol biosynthesis', 'porphyrin and chlorophyll metabolism', 'glyoxylate and dicarboxylate metabolism', 'Metabolic pathways' and 'Biosynthesis of secondary metabolites'. Substantial numbers of genes/proteins involved in signaling pathway, protein turnover, nutrient transport, hormone action, cell rescue and defense were identified in OM symbiosis specifically.

### Validation of DEG profiling with qRT-PCR

Using additional biological samples of ML01-, ZH3A-3- and MZ- colonized roots in comparison to mock-inoculated control roots, we performed real-time RT-PCR to verify the digital expression profiles of 26 genes corresponding to a range of functional categories and regulation patterns in the *C. hybridum*- beneficial fungi interactions. The Pearson correlation coefficient was calculated by SPSS to assess the correlation between different platforms. Overall, the qRT-PCR values were highly correlated with the RNA-seq results, which confirmed the general expression pattern for all the selected genes in three treatments (Table 3 and Additional file 17: Figure S10, R^2^ = 0.847, correlation was significant at the 0.01 level).

**Table 3:** **Comparison of the expression profiles of 26 genes by RNA-seq and quantitative real-time RT-PCR**

Gene ID	Annotation	M/CK (log _2_fold)		Z/CK (log _2_fold)		MZ/CK (log _2_fold)	
		RNA-seq	qRT-PCR	RNA-seq	qRT-PCR	RNA-seq	qRT-PCR
Unigene 1724_All	ethylene receptor homolog2	1.72	1.59 ± 0.24	1.19	0.73 ± 0.09	1.87	1.51 ± 0.05
Unigene 1869_All	LysM domain receptor-like kinase 3-like	4.26	4.29 ± 0.06	0.51	0.98 ± 0.19	3.97	3.85 ± 0.03
Unigene 2625_All	calmodulin binding protein, putative	1.19	0.95 ± 0.16	0.87	0.38 ± 0.15	1.30	0.64 ± 0.10
Unigene 4130_All	sugar transporter ERD6-like 7-like	1.74	1.70 ± 0.21	0.84	1.01 ± 0.05	2.03	1.63 ± 0.38
Unigene 4239_All	boron transporter 2	0.96	0.58 ± 0.25	-2.17	-3.07 ± 0.06	0.61	0.27 ± 0.39
Unigene 6086_All	inorganic phosphate transporter, putative	3.01	2.81 ± 0.34	2.49	2.90 ± 0.26	3.40	3.06 ± 0.10
Unigene 8895_All	Xyloglucan endotransglucosylase/hydrolase	2.03	0.58 ± 0.66	2.55	1.74 ± 0.49	1.89	-0.07 ± 0.38
Unigene 10786_All	equilibrative nucleoside transporter	3.95	4.71 ± 0.11	-0.21	0.42 ± 0.08	3.94	4.41 ± 0.05
Unigene 12197_All	ethylene-responsive element binding factor 4	2.33	1.83 ± 0.53	3.12	2.72 ± 0.39	3.37	2.74 ± 0.11
Unigene 13078_All	glutathione peroxidase	4.20	2.62 ± 0.15	1.67	1.04 ± 0.27	3.95	2.45 ± 0.06
Unigene 13219_All	transmembrane copper transporter	-6.93	-1.01 ± 0.03	8.05	8.15 ± 0.44	6.51	4.65 ± 0.06
Unigene 13639_All	ATPase 7, plasma membrane-type-like	4.24	4.39 ± 0.09	0.52	0.32 ± 0.11	3.94	3.81 ± 0.09
Unigene 14415_All	major facilitator family protein	-2.13	-1.55 ± 0.50	-0.97	-1.13 ± 0.86	-2.36	-1.91 ± 0.50
Unigene 15955_All	sorbitol transporter	2.32	2.86 ± 0.38	0.87	1.54 ± 0.21	2.27	2.20 ± 0.07
Unigene 13573_All	cellulose synthase A catalytic subunit 4 [UDP-forming]-like	4.76	7.26 ± 0.91	4.94	7.61 ± 0.87	4.67	6.50 ± 0.89
Unigene 2569_All	transcription factor, putative	2.18	2.68 ± 0.30	0.00	0.38 ± 0.20	2.00	2.41 ± 0.32
Unigene 14999_All	NAC domain-containing protein 43-like	4.19	3.73 ± 0.36	4.60	4.53 ± 0.32	4.32	3.09 ± 0.18
Unigene 16297_All	CBL-interacting serine/threonine-protein kinase 11	1.80	1.34 ± 0.35	1.61	1.80 ± 0.18	2.17	1.55 ± 0.35
Unigene 10727_All	zinc finger CCCH domain-containing protein 66-like	2.22	2.07 ± 0.24	3.09	2.82 ± 0.04	3.53	2.87 ± 0.01
Unigene 15958_All	chitinase 4-like	20.38	13.75 ± 0.15	0.00	-0.04 ± 0.34	20.09	13.40 ± 0.21
Unigene 4429_All	laccase-4-like	2.35	2.35 ± 0.29	2.81	3.38 ± 0.06	1.96	1.81 ± 0.19
Unigene 11039_All	auxin efflux facilitator PIN1	3.42	3.27 ± 0.39	3.74	2.84 ± 0.07	3.77	3.00 ± 0.25
Unigene 15779_All	Xylem serine proteinase 1 precursor, putative	1.54	2.09 ± 0.30	2.40	3.21 ± 0.13	1.79	2.32 ± 0.20
Unigene 2376_All	peptide transporter PTR3-A-like	2.92	2.38 ± 0.37	-1.01	-1.44 ± 0.45	2.79	1.70 ± 0.14
Unigene 2386_All	oligopeptide transporter 1-like	4.51	4.90 ± 0.49	1.66	2.51 ± 0.46	4.57	4.86 ± 0.49
Unigene 13170_All	gibberellin 20-oxidase	1.66	2.13 ± 0.05	0.03	0.45 ± 0.16	1.14	1.00 ± 0.27

Fold changes of selected *C. hybridum* trasncripts (±standard deviation) were logarithmic transformed which were determined by RNA-seq and qRT-PCR in root tissues treated with isolates ML01 (M) , ZH3A-3 (Z) and ML01+ ZH3A-3 (MZ) at 15 dpi as compared to mock-inoculated control (CK).

Base on the qRT-PCR results, we further analyzed the functional distribution of all the confirmed genes. The function of co-regulated genes in three treatments included signaling (CBL-interacting serine/threonine-protein kinase 11), membrane proliferation (cellulose synthase A catalytic subunit 4), scavenging of reactive oxygen species (glutathione peroxidase), general stress- responsive (laccase-4-like), nutrient transport (inorganic phosphate transporter, major facilitator family protein, oligopeptide transporter 1-like), protein processing (xylem serine proteinase 1 precursor), transcription factor (ethylene-responsive element binding factor 4, NAC domain-containing protein 43-like, zinc finger CCCH domain-containing protein 66-like) and hormone transport (auxin efflux facilitator PIN1). DEGs derived exclusively from OM roots were assigned to signaling (LysM domain receptor-like kinase 3-like), transport (equilibrative nucleoside transporter, ATPase 7, plasma membrane-type-like, peptide transporter PTR3-A-like), transcription factor (Unigene 2569_All), cell defense (chitinase 4-like) and hormone metabolism (gibberellin 20-oxidase).Two genes showed special expression pattern in response to ZH3A-3 treatment, including Unigene 4239_All (putative boron transporter 2) and Unigene 13219_All (putative trans-membrane copper transporter).

## Discussion

### Some different effects of mycorrhizal and non-mycorrhizal fungi on the growth of *C. hybridum*plantlets

This study demonstrated that the vegetative growth of *C. hybridum* plantlets could be improved by orchid mycorrhizal fungus ML01 and/or non-mycorrhizal isolate ZH3A-3. It is widely accepted that *Tulasnella*-like *Rhizoctonia* formed typical mycorrhizae with orchids and improved their germination and their seedlings’ growth [40, 41]. Consistent with these studies, ML01 formed typical pelotons in host root cortex and significantly improved the total fresh weight (FW) and dry biomass (DW) of *C. hybridum* plantlets compared to the control. However, we observed that the roots of ML01- treated plantlets did not elongate obviously as the co-cultivation proceeded, which might be due to rapid acidification of the co-cultivation media (our unpublished data). It was thus inferred ML01 increased the total dry biomass of orchid plantlets through improving the absorption of mineral nutrient. Non-mycorrhizal fungus ZH3A-3 alone showed similar promoting effects on total FW and DW of *Cymbidium* plantlets to that of MZ treatment at 45 dpi (Table 1), but we also observed that individual ZH3A-3- treated orchids initiated less new roots than that of dual- inoculated plantlets, and their leaves turned into light green after a prolonged co-cultivation. Isolate ZH3A-3 always co-existed with *Tulasnella*-like *Rhizoctonia* in wild orchid roots and the hyphae of isolate ZH3A-3 mainly spread in velamen cells and could not form pelotons in the cortex cells of *Cymbidium* roots. It was also revealed that the effects of isolate ZH3A-3 and another OMF isolate (CL01) on hosts’ uptake of mineral nutrient were complementary in our recent report [8]. Thus, we designated ZH3A-3 as mycorrhizal-associated fungi.

### Functional analyses of differentially regulated genes by OM symbiosis

The establishment of OM symbiosis involves the mutual recognition of host and fungi, hypha penetrating the cell walls of root hairs or epidermis and then entering the cells of the cortical parenchma, forming characteristic and complex hyphal coils (pelotons) [2]. As in other endomycorrhizal systems, the plant cell membranes in the infected tissues remain intact at all times, excluding the hyphae from the cytoplasmic compartment, and an interfacial matrix is formed between the invading hyphae and the invaginating plant plasmalemma [5]. The fungi further spread either as a result of repeated colonization or by hyphae penetrating from cell to cell in the root cortex, and the cortex cells may be uncolonized or contain active pelotons or clumps of degenerating hyphae in different proportions. So, Rasmussen *et al.* [5] proposed that there co-existed necrotrophic and biotrophic phase in orchid mycorrhiza, and the former referred to peloton degradation and unilateral nutrient transfer by endocytosis, and the latter, mutual nutritional exchange [5]. A recent labeling study in *Spiranthes sinensis* clearly indicated that in the symbiotic protocorm, hyphal degeneration played a significant role in the mass transfer of the elements to plant cells, and fungal carbon and nitrogen was also detected to be transferred from live hyphae through the interface between the symbionts [42]. Owing to the non- synchronous process, the symbiotic root system often contains fungal structures in different developmental stages (contact, penetrating hyphae, intracellular hyphae, active and degenerating pelotons), and the functions of DEGs for OM symbiosis were widely distributed.

#### *Genes related to signaling*pathway

LysM-domain containing receptor-like kinases (LysM-RLK) were identified as receptors of acylated chitin (Nod factors) or chitin produced by plant interacting microbes and confirmed to be involved in signaling during nodule and AM development [13, 43, 44]. In this context, it is noteworthy that two genes (Unigene8696_All, Unigene 1869_All) predicted to encode LysM domain receptor-like kinases showed evident up-regulation in OM symbiosis. One of them, Unigene 1869_All, encodes a protein with 56% identity to LysM type receptor kinase LYS3 from *L. japonicus* (BAI79284). Its induction was validated by qRT-PCR, which measured 16-fold up-regulation in the mycorrhizal roots. This was consistent with the notion that symbiosis signaling seems to occur throughout the process of root colonization by a symbiont and occurs in parallel to additional signaling mechanisms that require the LysM receptor-like kinases [45].

Ca^2+^ spiking in the nucleus and perinuclear region of root hair cells has been documented as one of the earliest cellular responses after the perception of symbionts by host plants [46]. Our digital analysis also revealed many transcripts associated with Ca^2+^ signals, as annexin and calcium-binding EF hand family proteins were co-regulated by three treatments. But we also detected some transcripts in this group were specifically regulated by OM fungi, including five genes encoding calcium-binding proteins (Unigene4048_All, Unigene6332_All, Unigene11025_All, Unigene3685_All, Unigene3072_All), two genes encoding homologues of calcium-dependent protein kinase (CDPK) (Unigene6383_All, Unigene8933_All), one cyclic nucleotide-gated ion channel protein (Unigene5311_All), and one gene (Unigene6051_All), encoding a homologue of DMI2 nodulation receptor kinase from *M. truncatula*.

Another interesting finding was that two transcripts (Unigene10121_All and Unigene456_All, up-regulated more than 2-fold) encoding putative GRAS family protein with equal 44% identity to LjNSP1 (nodulation-signaling pathway 1 protein) from *L. japonicas*, and one gene (Unigene7236_All, down-regulated more than 2-fold), which encoded GRAS family transcription factor with 56% identity to NSP2 (nodulation-signaling pathway 2 protein) from *M. truncatula*, were also specifically regulated by OM symbiosis. NSP1 and NSP2 are required for rhizobium-specific gene expression whereas only NSP2 might be required for mycorrhizal signaling and NSP1 is replaced with required for arbuscular mycorrhization 1 (RAM1) to drive expression of mycorrhiza-specific genes [45].

#### Genes involved in cellular organization and protein processing

A substantial number of genes related to cell wall modification were found to be co-regulated in three treatments, which encoded probable xyloglucan endotransglucosylase, beta-D-xylosidase or cellulose synthase. Two of these were confirmed by qRT-PCR. Unigene 8895_All, encoding a protein with 75% identity to the xyloglucan endotransglucosylase (XET) of *Medicago trancatula*, was co-induced more than 4-fold. Another gene (Unigene 13573_All) was strongly up-regulated more than 32-fold upon symbiosis (Table 3). The translated protein shares 92% identity to cellulose synthase A (CesA) catalytic subunit of *Vitis vinifera*. CesA proteins are part of the cellulose synthase complex in higher plants located in the plasma membrane [47]. Guether *et al.* [11] also confirmed that two transcripts of *Lotus*, *LjCel1* and *LjCesA*, accumulated specifically in the arbusculated cells and functioned in cell expansion during arbuscule development [11]. The strongly up-regulated transcripts of putative cellulose synthase might be involved in plant cell wall reinforcement during fungal colonization.

Similar to AM fungi, orchid mycorrhizal fungi penetrate the plant cell wall but not the plasma membrane. As the colonization proceeds, the host plasma membrane invaginates and the infected cells often contain numerous mitochondria, ribosomes and profusely developed ER. A large number of sequences that were specifically induced by OM formation were assigned to protein metabolism and turnover, membrane dynamics and cell wall synthesis. This group of OM specifically induced genes in *Cymbidium* were thought to be homologues associated with AM and ectomycorrhizal symbioses [11].

Besides the co-regulated DEGS among three treatments, OM symbiosis also specifically induced a high number of transcripts encoding cell wall-degrading enzymes, including five glucan endo-1,3-beta-glucosidase, one pectinesterase 3 and one xyloglucan endotransglucosylase/hydrolase protein. Concomitantly, three extensin- like proteins (Unigene11713_All, Unigene13380_All, Unigene16702_All), and three putative glycosyltransferases (Unigene12916_All, up-regulated more than 300-fold; Unigene12916_All and Unigene9981_All, up-regulated more than 4-fold) involved in cell wall reinforcement were also found in our dataset. Overall, this indicates an importance of wall- strength maintenance in OM symbiosis. With regard to membrane proliferation, two homologues of *A. thaliana* syntaxin SYP 132, Unigene10103_All and Unigene7281_All, were exclusively induced 16- and 6- fold by mycorrhizal symbiosis. Syntaxin belong to the superfamily of the SNARE proteins that play key roles in membrane fusion events during vesicle trafficking. In AM symbiosis, a gene (Ljwgs_016013.1) encoding a protein with a 60% identity to the *A. thaliana* syntaxin SYP 132 also showed seven-fold up-regulation and was suggested to facilitate plant/fungal compatibility in mycorrhizal *Lotus* roots [11].

Protein metabolism and processing seems to be a major process in mycorrhizal symbiosis [11, 48, 49]. Most drastically up OM- regulated genes in the present study were involved in protein synthesis and processing (Additional file 11: Tables S10 and S11). Concerning protein synthesis, the strong induction of different ribosomal proteins in response to OM fungi provided evidence. A number of transcripts associated with protein turnover were also detected in our analysis, including three subtilisin-like proteases (Unigene7254_All and Unigene16547_All, up-regulated more than 1000-fold; Unigene10817_All, up-regulated more than 2000-fold), three serine carboxypeptidase (Unigene4706_All, up-regulated more than 1500- fold; Unigene12600_All, up-regulated more than 300-fold; Unigene5600_All, up-regulated more than 6-fold), and also some peptidase (Unigene15372_All, up-regulated more than 320 fold; Unigene2916_All, up-regulated more than 40-fold; Unigene11542_All, up-regulated more than 4-fold). Some transcripts associated with correct protein folding (Unigene4797_All, Unigene7897_All) and degradation of mis-folded protein (Unigene11189_All, Unigene11236_All, Unigene15698_All, Unigene7179_All, Unigene 6288_All) were also identified as specifically up-regulated in OM symbiosis. At the same time, a transcript of putative serine-type endopeptidase inhibitor, Unigene16319_All, accumulated more than 40-fold in the mycorrhizal roots compared with controls, which might fine-tune protein processing.

#### Transport-related genes

Phosphate transfer to the adult orchids via mycorrhizal fungi was well documented, just as in other mycorrhizal systems. Recently, labeling experiments also directly demonstrated that the fully autotrophic orchid *Goodyera repens* acquires carbon, nitrogen and phosphorous from its fungal partner [9, 10, 50]. Notably, *G. repens* also transfers significant amounts of photosynthate (likely greater than 3% of its photosynthetic carbon) back to its *Ceratobasidium* mycobiont – the first direct demonstration of a net carbon flow from orchid to fungus [9, 10]. *Tulasnella*-like *Rhizoctonia* were also confirmed to improve the plant growth of *C. spp*. by enhancing nutrient acquisition [8].

**Figure 6:**
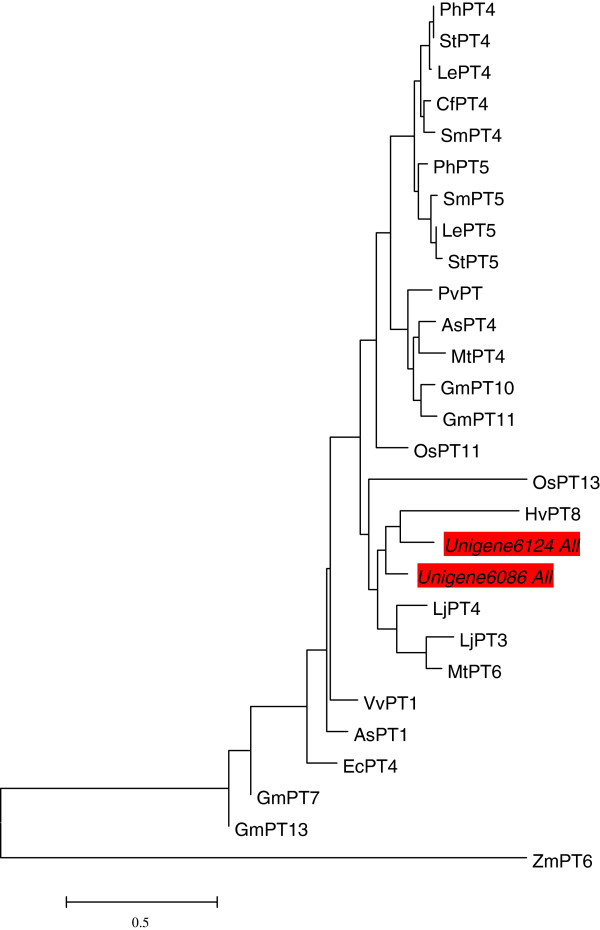
**Phylogenetic tree for the amino acid sequences of plant phosphate transporters (PiTs) described as mycorrhiza-specific or -induced transporters, and two putative PiTs of** ***C. hybridum*** **(Unigene6086_All, Unigene6124_All) in this study were highlighted in red color.** The dendrogram was generated by Mega 4.0 software using ClustlW for the alignment and the neighbor-joining method for the construction of phylogeny. The plant Pi-transporters accession numbers could be obtained in [49].

In our global profiling analysis, 15 genes associated with membrane transport were identified as co-regulated in three treatments. Among them, 7 genes involved in nutrient transport were co-induced in symbiotic roots, including two inorganic phosphate transporters (Unigene6086_All, Unigene6124_All), a plasma membrane ATPase 4 (Unigene6958_All), a BCS1 protein precursor-like (AAA ATPase chaperone, Unigene14840_All) a sorbitol transporter (Unigene15955_All), an oligopeptide transporter 1-like (Unigene2386_All) and a magnesium transporter (Unigene11188_All), whereas 8 genes were co-repressed at this time point which encoded two putative copper-transporting ATPase, a copper transporter, a probable nitrite transporter, a sulfate transporter 3.1, an inorganic phosphate transporter 2–1, chloroplastic, a probable sodium-coupled neutral amino acid transporter 6 and a member of major facilitator family. Of these, the expression pattern of five genes was further verified by qRT-PCR. The first of these, Unigene6086_All, was co-induced more than 5-fold, which encodes an inorganic phosphate transporter with 81% similarity to inorganic phosphate transporter 1–4 of *Arabidopsis thaliana* and a shared 78% identity to inorganic phosphate transporter from *Lotus japonicus* (LjPT3, LjPT4) and *M. truncatula* (MtPT6). Both deduced amino acid sequences of two up-regulated inorganic phosphate transporter genes (Unigene6086_All, Unigene6124_All) in our digital profiling have similarity to reported mycorrhiza-specific or -induced phosphate transporters [51] (Figure 6). The second gene, Unigene2386_All, which was up-regulated between 4- and 16- fold in the symbiotic process, encodes a protein with 63% identity to *V. vinifera* oligopeptide transporter 1-like. Another two co-induced genes (Unigene15955_All, Unigene4130_All) were involved in carbohydrate transmembrane transport and further identified by qRT-PCR (Table 3). Unigene15955_All encodes a putative sorbitol transporter with 71% similarity to sorbitol transporter of *Zea mays* and Unigene4130_All encodes a transporter with 60% identity to sugar transporter ERD6-like 7 of *A. thaliana*. Finally, a member of a major facilitator family (Unigene14415_All) was confirmed to be repressed more than 4-fold in symbiotic roots and its translated sequence shared 68% similarity to a major facilitator family protein of *A. thaliana*. In general, the regulation of a number of transporter genes supported the notion that extensive nutrient exchange was involved in symbiosis of the host and beneficial fungi.

We also identified a substantial proportion of genes involved in transport which were specifically regulated in mycorrhizal roots. These included three transcripts (Unigene11510_All, up-regulated 100 fold; Unigene13639_All, up-regulated 15 fold; Unigene2212_All, up-regulated 32 fold) encoding putative plant plasma membrane proton ATPases. The amino acid sequence of the encoded protein by Unigene11510_All shares 77% identity with the AM-specific Mtha1in *M. truncatula.* The regulation of another transcript (Unigene13639_All) was validated by qRT-PCR (Table 3) and its translated sequence shared 70% similarity to H^+^-ATPase 7 from *A. thaliana*. In OM, like in AM and ECM, nutrients from symbiotic hyphae separated at the apoplastic space have to go through several membrane barriers before being assimilated by the partner's cells, so the activated plant plasma membrane proton ATPase might be a sign of accelerated membrane transport. The other two genes (Unigene13137_All, up-regulated 7 fold; Unigene3152_All, up-regulated 2 fold) were also identified as OM-specific which encoded a putative copper-exporting ATPase and a plasma membrane calcium-transporting ATPase, respectively.

Another group of mycorrhizal-induced genes identified was associated with nitrogen transport. Eight genes of peptide transporter (PTR) were found to be exclusively up-regulated between two-fold and more than 300-fold compared with controls. The strongest up-regulated PTR (Unigene4918_All), encoding a homologue of peptide transporter PTR1 of *M. truncatula* with 62% identity, showed none expression level in the control and CyEX22 samples. The second putative PTR (Unigene8412_All) was 300-fold up regulated in mycorrhizal roots at 15 dpi and had a 76% similarity to a member of oligopeptide transporter family (OPT) from *P. trichocarpa*. Quantitative RT-PCR analysis confirmed that one transcript (Unigene2376_All) was up-regulated more than 7-fold in OM symbiosis whereas down regulated two-fold in ZH3A-3-treated orchid roots. Besides these PTRS, two putative amino acid transporters were also detected to be induced by mycorrhizal formation. A homology search revealed 70% identity between the translated amino acid sequence of Unigene3142_All and cationic amino acid transporter 5 (CAT5) of *A. thaliana*. The second gene, Unigene404_All, encodes a protein with 81% identity to LHT1 (lysine histidine transporter 1) of *A. thaliana* (thale cress), which was regulated by diverse factors. Our data supported the notion that OMF played an important role in improving N acquisition of host plants [9, 27].

Two putative monosaccharide transporter genes (Unigene12841_All, Unigene4472_All) were also found to be induced in OM mycorrhizal roots. The closest homologue of translated sequence by Unigene12841_All in *A. thaliana* is sugar-proton symporter PLT5 (AtPLT5), with 71% identity at amino acid level. Another gene, Unigene4472_All, encoded a homologue with 70% similarity to a tonoplast monosaccharide transporter 2 (TMT2) in *A. thaliana* and TMT1/2 probably represents a family of proton-coupled antiporters capable of high-capacity loading of glucose and sucrose into the vacuole. Gaude *et al.* [48] also demonstrated that two putative sucrose transporters and one hexose transporter were induced in non-colonized cortical cells of mycorrhizal roots compared to the cortical cells of non-mycorrhizal roots, and suggested they might be involved in the uptake of carbohydrate from the apoplast into cells in the vicinity of hyphae in arbuscular mycorrhizal roots or functioned in the proton-coupled uptake of carbohydrate into the cytoplasm from the vacuole [48]. This raises a compelling question of what is the exact function of these two putative monosaccharide transporters in our study. Finally, a gene encoding a putative potassium transporter (Unigene14628_All) was also detected to be 6-fold up-regulated in mycorrhizal roots and shared 50% identity at the amino acid level with the potassium transporter 1 of *A. thaliana.*

In addition to a number of up-regulated genes probably involved in nutrient transport, ATP binding cassette (ABC) transporter genes, which are unlikely associated with direct nutrient transport, were also identified to be regulated in mycorrhizal roots. The strongest up-regulated gene of ABC transporter family (Unigene5835_All, up-regulated nearly 1,000-fold) encoded a protein with 66% identity to PDR3 ABC transporter of *Oryza sativa*, which was regulated by many abiotic and biotic factors. Recently, two plant half-size ATP binding cassette (ABC) transporter proteins (STR/STR2) were identified that are essential for AM symbiosis [52] and one full-size ABC transporter gene *MtABCB1* (Medtr8g025810) showed strongly increased transcript levels in arbuscule-containing (ARB) cells and also in adjacent cells [48]. These ABC transporter proteins may be involved in the transport of signaling between plant cells during AM symbiosis [48]. Here, these identified ABC transporter genes showing different expression patterns in orchid mycorrhizal roots may be important for OM symbiosis.

OM-activated transcripts also included components associated with vesicle-mediated transport, which might involve in the membrane biogenesis for an intracellular accommodation of fungal structures. The translated sequence of Unigene7513_All (up-regulated more than 3-fold) shares 70% identity with VAMP7B (vesicle- associated membrane protein 7B) in *A. lyrata subsp. lyrata*. Another gene, Unigene11358_All, encoding a putative clathrin assembly protein*,* was up-regulated more than 8-fold in mycorrhizal roots.

#### Defense -related and phytohormone regulation

Oxidative burst belongs to early events of plant responses to compatible and incompatible fungi. The success of beneficial microbes to colonize plant roots depends on their ability to manipulate immune responses of the host. This was verified by the findings of transcripts associated with scavenging of reactive oxygen species (ROS), such as glutathione peroxidase, probable glutathione S-transferase (GSTF1) and germin-like protein (Unigene6491_All, up-regulated more than 37-fold; Unigene5007_All, up-regulated more than 25-fold; Unigene4304_All, up-regulated more than 4-fold), accumulating in the symbiotic roots. One of these, Unigene13078_All, encodes a protein with 77% identity to the glutathione peroxidase of *Populus trichocarpa* and was further validated by qRT-PCR.

Concomitantly, many transcripts encoding general 'stress- responsive' proteins (e.g. laccase-4-like; probable pectinesterase/pectinesterase inhibitor 34 (Unigene11474_All); bibenzyl synthase (Unigene1160_All, Unigene1836_All, Unigene4746_All) were identified to be induced in these symbiotic process. The enzymes in the pathway towards fungicidal phytoalexin synthesis are produced as a general feature in orchids. Reinecke [53] have reported that the activity of benzyl synthase, catalyzing the biosynthesis of bibenzyls and 9, 10-dihydrophenanthrenes of *Bletilla striata* rhizomes, increased substantially upon fungal infection [53]. Another interesting finding was that two transcripts encoding programmed cell death protein 4 (PDCD4, Unigene3283_All and Unigene14957_All) were down regulated collectively in three treatments. PDCD4, a suppressor of gene transcription and translation, plays a crucial inhibitory role in several types of human tumors through inhibiting autophagy in multiple cell types both *in vitro* and *in vivo* [54]. Down- regulation of these homologues of PDCD4 may promote the symbiotic process through suppressing plant defense. Furthermore, four transcripts (Unigene4420_All, Unigene8976_All, Unigene8566_All, Unigene13020_All), encoding homologues to key molecular regulators in two-component systems (TCSs) which play important roles in responses to environmental stress stimuli [55], were found to be down regulated regardless of the inoculated fungi species, highlighting the shared strategy by different beneficial fungi in the symbiosis.

Apart from these shared DEGs, three chitinase genes (Unigene15958_All, Unigene16629_All, Unigene8905_All) were exclusively induced by OM fungi and the specific expression pattern of Unigene 15958_All was confirmed by qRT-PCR. The translated sequence of Unigene 15958_All shared 59% identity with the chitinase 4-like of *Fragaria vesca*. Chitinases hydrolyze β-1,4-glycosidic bonds and can be induced in pathogenic interactions. But the expression of Unigene 15958_All was not induced by a pathogenic *Fusarium* isolate (our unpublished data). Unigene8905_All encoded a protein with 55% identity to class III chitinase of *M. truncatula* and class III chitinases were found to be expressed predominantly in the arbuscule-containing cells [14, 56], where they may suppress plant defense by the reduction of chitin-like elicitors during the formation of functional symbiotic interfaces [57]. The similar role of class III chitinase Sv*Chit3* in OM was recently suggested by Perotto [19]. Thus, these up-regulated chitinases are probably involved in the lysis of fungal cell wall and balancing the symbiosis.

Another notable OM-regulated group was mannose-specific lectins (Unigene4318_All, Unigene529_All, Unigene14222_All). Among these, Unigene4318_All and Unigene529_All showed high-level expression levels in mycorrhizal roots with little expression in the un-inoculated control and ZH3A-3 treatments. Antifungal activity against a range of phytopathogenic fungi has been demonstrated for some of mannose-specific lectins both *in vitro* and in transgenic plants, and they were thus proposed to be part of defense mechanisms against the invading fungus. The strong up-regulation of *SvLect3* and *SvLect5* in response to mycorrhizal fungi *T. calospora in S. vomeracea* protocorm was recently confirmed by qRT-PCR and this was suggested to be part of a mechanism to limit fungal growth in the mycorrhizal protocorm [19]. However, another mannose-specific lectin gene, Unigene14222_All, with 49905.50 RPKM in control roots, was specifically down-regulated by OM fungi. Owing to the multi-functions of plant lectins, the role of these lectins in the orchid mycorrhizal process remains to be established.

Among plant hormones, ethylene (ET), salicylic acid (SA), abscisic acid (ABA), and jasmonic acid (JA) are known to be key elements in fine-tuning the plant defense response during interaction with other organisms [58, 59]. The inhibitory effect of exogenous ethylene in AM development has been documented and the colonization of host tissues by mutualistic AM fungi such as *Rhizophagus irregularis* (formerly *Glomus intraradices*), ectomycorrhizal fungus *Laccaria bicolor*, or endophytic fungus *Piriformospora indica* was inhibited by ET [60]. However, ET signaling is still essential for nodule formation in *Sesbania rostrata* and in the initial stages of *P. indica* colonization [61]. Because ethylene-responsive transcription factors (ERF) can function as transcriptional activators and repressors, they are candidates for establishing a balanced defense response to the fungus without preventing growth and development in AM symbiosis [62]. Valadares [20] also suggested the role of ethylene in OM regulation when compared the gene expression pattern of *O. maculata* mycorrhizal roots in comparison to non-mycorrhizal controls [22]. In our global expression profiling, eight transcription factors (TFs) related to ethylene signaling (Table 4) were found to accumulate in all the symbiotic roots and four genes among these were specifically induced in OM roots. Among these, the induction of two transcripts (Unigene1724_All, Unigene12197_All) was further validated by by qRT-PCR. The translated sequence of Unigene1724_All showed 63% similarity to the ethylene receptor homolog 2 (ETR2) of *Zea mays* with two-fold induction. Unigene12197_All, encoding a protein with 45% similarity to ethylene-responsive element binding factor 4 (ERF4) of *G. max,* was induced more than 5- fold in the symbiotic roots. As with Plett *et al.* [60], the induction of these TFs is probably an adaptive response by the plant so that its growth and vigor are not compromised by the fungus in the late stage of symbiosis [60].

**Table 4:** **Transcription factors among beneficial fungi- induced genes**

TF family or domain	Induced	Repressed	Number of genes
AP2-ERF	Unigene627_All*, Unigene6402_All*, Unigene12197_All*⊕, Unigene103_All*, Unigene1545_All, Unigene10693_All, Unigene13239_All, Unigene13630_All		4* (4)
WRKY	Unigene2569_All ⊕ (WRKY19), Unigene13120_All*(WRKY40), Unigene8345_All*(WRKY5), Unigene8346_All*(WRKY5), Unigene3004_All*(WRKY39), Unigene4511_All*(WRKY43), Unigene1640_All(WRKY17)	Unigene1887_All (WRKY23)	5* (3)
Myb	Unigene10890_All*, Unigene13524_All*, Unigene14312_All*, Unigene2947_All	Unigene12533_All, Unigene16161_All*, Unigene1441_All*, Unigene14070_All*	6* (2)
bHLH	Unigene4212_All*, Unigene2945_All*, Unigene13683_All*, Unigene14108_All*	Unigene1570_All*, Unigene15208_All*, Unigene12890_All,	6* (1)
HOX	Unigene11426_All*, Unigene888_All,	Unigene9904_All, Unigene7407_All*, Unigene14238_All	2* (3)
NAC	Unigene14999_All*⊕, Unigene8664_All*, Unigene16437_All*, Unigene11336_All*	Unigene5046_All*	5*
ARF	Unigene10085_All (ARF9), Unigene9466_All (ARF9)		(2)
GRAS	Unigene2120_All(SC)*, Unigene10121_All, Unigene4453_All (scarecrow-like), Unigene6482_All (SC), Unigene13312_All (SC), Unigene15630_All, Unigene456_All		1* (6)
BTB/POZ	Unigene2160_All, Unigene351_All, Unigene352_All	Unigene11206_All	(4)
C3H	Unigene156_All, Unigene209_All*, Unigene10727_All*⊕	Unigene16285_All, Unigene81_All	2* (3)
GATA	Unigene1952_All*, Unigene10179_All*	Unigene1691_All, Unigene10219_All*	3* (1)
C2H2	Unigene2954_All*,	Unigene2352_All*	2*
dof	Unigene3028_All*,		1*
A20AN1	Unigene2954_All*, Unigene10730_All		1* (1)
CO-like		Unigene4931_All	(1)
Hsf	Unigene13648_All*, Unigene4980_All*		2*
Trihelix		Unigene2892_All	(1)
ASG		Unigene3840_All	(1)
WD	Unigene4225_All		(1)
MADs	Unigene4681_All		(1)
MACPF	Unigene8197_All		(1)
BLH	Unigene8238_All		(1)
A-2b	Unigene13648_All*, Unigene4980_All		1* (1)
UNE	Unigene4910_All	Unigene10862_All*	1* (1)
rf	Unigene14563_All*		1*
MYC	Unigene15000_All*		1*
RAX	Unigene1589_All*		1*
NF-YC	Unigene16018_All*		1*
bZIP	Unigene4820_All,		(1)

Lists of TFs in this table included specifically regulated TFs in OM formation and * co-regulated genes in three treatments; ⊕qPCR validation; number in parenthesis referred to the gene number regulated by OM formation.

ABA plays a crucial role in biotic and abiotic stress responses. Analysis of AM colonization in the *sitiens* ABA-deficient tomato mutant showed that ABA was necessary in order to complete arbuscule formation and its functionality and to promote sustained colonization of the plant root [63]. Subsequent work supported the notion that ABA deficiency negatively regulates mycorrhizal intensity/hyphal colonization directly or indirectly through increasing ethylene levels [64–66]. Accumulation of the transcripts related to ABA de novo biosynthesis was documented in AM roots [49] and OM green protocorms [20]. At the same time, genes associated with ABA catabolism also showed increased transcript levels in AM roots[49] and OM roots [22]. In our study, 4 transcripts related to ABA biosynthesis: 9-cis-epoxycarotenoid dioxygenase (NCED) (Unigene1444_All, Unigene2412_All) and zeaxanthin epoxidase (ZXE) (Unigene11284_All, Unigene13062_All), were co-suppressed in symbiotic roots. ABA deficiency might be involved in restriction of fungal spread in orchid symbiotic roots.

Jasmonic acid has long been implicated in the plant’s systemic response to pathogen attack [67]. While experimental results sometimes appear contradictory, most authors agree that jasmonate signalling is important for AM colonization and development [68]. Plett [60] revealed that in the interaction between *L. bicolor* and poplar, ethylene and jasmonic acid showed extensive transcriptional cross-talk and functioned as negative modulators during mutualistic symbiosis [60]. Jasmonates belong to a diverse class of lipid metabolites known as oxylipins and the key genes in the biosynthesis of oxylipins were commonly induced in mycorrhizal roots [59]. Consistent with these studies, the induction of 6 lipoxygenase (LOXs) genes in our study suggest the involvement of the oxylipin pathway in regulation of OM symbiosis. In plants, there are two main branches of the oxylipin pathway, determined by two different types of lipoxygenases (LOXs), 9-LOX and 13-LOXs. The 13-LOX pathway leads to the biosynthesis of JA and derivatives, and the relevance of the 9-LOX pathway in plant interactions with nematodes and pathogens has also been demonstrated recently [69, 70]. Further research is required to elucidate the exact role of these up-regulated LOXs in long-term balance of the OM symbiosis.

Apart from the potential roles for ET, ABA and JA in OM symbiosis, three transcripts associated with gibberellin (GAs) biosynthesis were also increased in abundance in orchid mycorrhizal roots: two genes (Unigene11581_All, Unigene3993_All) encoding gibberellin 3-oxidase (GA3ox) and another (Unigene13170_All) encoding GA 20-oxidase (GA20ox). The translated sequence of Unigene13170_All shares 77% identity to gibberellin 20-oxidase of *Triticum aestivum* and its induction was confirmed by qRT-PCR. The product of GA3ox and GA20ox activates GA, suggesting that GA synthesis is essential for OM formation. In barley- *P. indica* interaction, Schäfer [71] also observed that two barley mutants impaired in GA synthesis or perception, reduced colonization by *P. indica*. Similarly, homologues of genes involved in the GA biosynthesis were already identified as mycorrhizal-responsive in tomato plants at the mature (50 dpi) phase of the interaction [66]. Foo [68] reported that biologically active gibberellins suppress arbuscule formation in pea roots, and gibberellin signaling DELLA proteins are essential for this response. Owing to extensive transcriptional cross-talk between GA signaling and SA/JA responses, the GA-regulated defense response might be critical for establishing these long-term mutualisms.

#### Transcription factors (TF)

As detailed above, three treatments initiated common stress responses for symbiotic roots, so the majority of co-regulated TFs encoded AP2 domain TFs and ethylene-responsive TFs (ERF), WRKY TFs, Myb- family TFs, basic helix-loop-helix (bHLH) TFs and zinc-finger TFs (Table 4). DNA-binding WRKY, AP2-domain and ERF TFs are generally involved during pathogen-related stress responses [48, 61]. The co-regulated TFs may also be involved in root developmental process, as are MYB family, ERF and NAC family [72].

We also detected GRAS family and ARF TFs specifically induced by OM formation. Four transcripts (Unigene4453_All, Unigene6482_All, Unigene13312_All, Unigene15630_All) encoding for GRAS members were putative SCARECROW (SCR) protein encoding genes, considered to be genetic determinants of root identity. Previous studies also confirmed that members of the GRAS family are essential for nodule development [11] and they may be important for the regulation of gene expression in AM roots of *M. truncatula*, *L. japonicus* and *S. lycopersicum* [11, 12, 49]. Two putative ARF transcripts were also identified to be up- regulated in orchid mycorrhizal roots. The induction of putative ARFs has also been found during AM symbiosis in maize, rice and *M. truncatula*, but not in *L. japonicus* [72]. And in AM interaction, mycorrhizal roots exhibit morphological characteristics such as an increase in the number of lateral/fine roots during early growth phases, similar to auxin-treated roots [49]. Recently, Sukumar (2012) and Fusconi [73] summarized a compelling supportive role for involvement of auxin pathways in beneficial plant–microbe interactions [73, 74]. Chutima [75] reported that orchid-associated fungi *Tulasnella* sp. produced high levels of indole-3- acetic acid (IAA) with all biological activities in culture medium supplemented with 2 mg/ml of L-tryptophan [75]. The IAA production by isolates ML01 was also identified in our research group (unpublished data).Furthermore, Unigene11039_All, predicted to translate a protein with 66% similarity to an auxin efflux facilitator SlPIN1 of *Solanum lycopersicum*, showed 15-fold up-regulation in the symbiotic roots. All these data point to the probable involvement of auxin signaling pathway in OM functionality.

### DEGs specifically regulated by non-mycorrhizal fungus ZH3A-3

The hyphae of non-mycorrhizal fungus ZH3A-3 colonized the velamen cells of orchid roots and individual inoculation significantly improved the root length and FW of *C. hybridum* plantlets. As detailed above, 845 genes were significantly regulated in *C. hybridum* roots inoculated with ZH3A-3 alone and most of these DEGs were shared with those of ML01 and/or MZ treatment. Apart from these shared DEGs, we also identified 143 genes were specifically regulated by ZH3A-3 treatment.

The largest number of up-regulated transcripts by ZH3A-3 treatment was associated with orchid root development. These genes are mainly related to hormonal balance and transcription regulation. Among plant phytohormones, auxin, cytokinin, and ethylene play important role for root development. Consistent with this, we detected the up-regulation of many corresponding genes, which encoded one cytokinin dehydrogenase (Unigene14465_All), two ERF (Unigene15166_All, Unigene7827_All), and three ABC transporter B family members (Unigene16320_All, Unigene5921_All, Unigene14693_All). Auxin plays a pivotal role in many plant- beneficial microbe interactions [74], and it is well established that higher auxin/cytokinin ratio determines the root initiation. A recent study also clearly demonstrated that *OsCKX4*, a cytokinin oxidase/dehydrogenase (CKX) family gene, integrates cytokinin and auxin signaling to control rice crown root formation positively [76]. So, the up-regulation of Unigene14465_All, which encodes a homolog with 69% identity to CKX of *A. thaliana*, may suggest its positive role in orchid- ZH3A-3 interaction. Polar auxin transport (PAT) is essential for establishment of auxin gradient that spatially defines the quiescent center (QC) and consequently the fate of neighboring stem [77]. ABC transporter B family members were probably involved in auxin acropetal or basipetal transport [78], thus, the up-regulation of three ABC transporter B family members appears to converge on the auxin pathway to modulate root development. As shown in Table 4, the putative MYB-TFs ranked the third largest number of the regulated TFs in the mycorrhizal symbiosis, and most were shared by three treatments, which suggested their essential role in response to symbiotic fungi. Recently, a convincing research supported that a putative MYB-like TF of *L. japonicus* (*LjMAMI*) which was AM-responsive may also have non-symbiotic functions: i.e. root growth [79]. In our study, two transcripts (Unigene2947_All and Unigene1589_All),encoding homologues of Myb family TFs of *O. sativa* Japonica Group and *A. thaliana* with 60%- 80% identity, were also detected to be up-regulated 4-fold by ZH3A-3.The translated sequence of Unigene2947_All also shared 46% similarity to *LjMAMI* at amino acid level. Another up-regulated TF related to root development was Unigene489_All (up-regulated 2-fold), which encodes a protein with 45% similarity to SCARECROW-like protein of *Z. mays*.

We also identified a substantial number of genes related to general disease resistance processes which were specifically up-regulated by ZH3A-3 treatment. These included 3 transcripts related to signaling (leucine-rich repeat transmembrane protein kinase), 5 transcripts associated with balance of reactive oxygen (respiratory burst oxidase homolog, class III peroxidase, L-ascorbate oxidase, monocopper oxidase-like protein), and 12 transcripts involved in secondary metabolism (phenylalanine ammonia-lyase, DnaJ homolog, disease resistance response protein, isoflavone 2'-hydroxylase, syringomycin biosynthesis enzyme). The increased activity of chitinases were generally found in nonspecific, broad-spectrum defenses, but we did not detect the transcript accumulation of chitinase genes in CyEX22.The fungal cell wall of isolate ZH3A-3 always ruptured as the cultivation proceeded (our unpublished data), which might be the reason. The activation of basal defense mechanisms of orchid plants by beneficial fungus ZH3A-3 did not affect the symbiotic effects, as described in [80].

As ZH3A-3 colonization induced dramatic structural changes in root cells and promoted the root growth of orchids, transcripts accumulated in the symbiotic roots were also related to carbohydrate and amino acid metabolism, protein synthesis and processing, and structural re-organization. Furthermore, a transcript (Unigene15068_All) involved in water transport was also up-regulated, which encodes a homolog of aquaporin PIP1-2 of *Z. mays* with 87% identity.

Taken together, we confirmed the findings which suggested that the development and metabolism of plant symbionts are generally driven by differential regulation of transcriptional regulators, signal transduction, and metabolic pathways, rather than by expression of symbiosis-specific genes [81].

### Detection of putative fungal genes in symbiotic roots

Owing to the distinct phylogenetic relationship between isolate ML01 and ZH3A-3, we could detect 163 possible fungal genes combining the annotation information of *de novo* assembled transcripts with the comparative expressed level in different symbiotic roots (Additional file 18: Tables S15 and S16). Of these 163 genes, 53 were probably genes from isolate ZH3A-3, and the rest, from ML01. The origin of putative ML01 genes was further confirmed by Blastn search against the *T. calospora* draft genome or *T. calospora* mycelium ESTs with an E value > 1e^-20^ (http://genome.jgi.doe.gov/pages/blast.jsf?db=Tulca1).

Transcripts from ML01 in the mycorrhizal roots were largely assigned to ribosomal proteins, enzymes that might be involved in plant cell wall degradation (1,4-beta-cellobiosidase A) and remodeling the fungal cell wall during growth and symbiosis (glycosyltransferase family 2 protein, chitin deacetylase, UDP-glucose pyrophosphorylase, endo-beta-mannanase 4). We also identified numerous transcripts encoding different proteins associated with the nutrient transport and assimilation, such as H^+^-transporting ATPase, phosphate transporter (Unigene5906_All), ammonium transporter (Unigene2660_All), amino acid and protein transporters, ZIP-like iron-zinc transporter, monosaccharide transporters (Unigene4644_All, Unigene12394_All) and MFS-type transporters (Unigene16198_All, Unigene2162_All). ABC-type Fe^3+^-siderophore transporter and other transporters most probably implicated in the detoxification process were also detected in the mycorrhizal roots. The translated amino acid sequence of Unigene5906_All shared 59-60% identity to high-affinity Pi- transporters from *G. versiforme* (GvePT) and *R. intraradices* (RinPT) (Additional file 19: Figure S11). The function of RinPT (also GinPT in references) has been suggested by Tisserant *et al.* [82] to be involved in stabilizing cooperation in the mycorrhizal symbiosis [82]. The transcript abundance of Unigene5906_All in CyEX23 was higher than that in CyEX21. So, this gene might be responsible for the better growth- promoting effect of MZ treatment than that of ML01 alone. Besides the transcripts in N and P assimilation pathway, iron-zinc transporter and ABC-type Fe^3+^-siderophore transporter co-existed in the mycorrhizal roots, which suggest an important role of some metals in OM functionality. Our previous study showed that isolate CL01, *Tulasnella*-like *Rhizoctonia*, formed typical mycorrhizae with orchids and enhanced the N, P, Zn, Fe contents of mycorrhzial plants [8]. The ITS sequence of ML01 shared 84% similarity to that of isolate CL01 and both functioned similarly in improving the growth and nutritional absorption of orchid plantlets (our unpublished data). However, there is a need to compare the differences in gene expression between the intraradical and extraradical mycelium so as to elucidate the role of these transcripts in OM symbiosis.

## Conclusions

Using a combination of RNA-seq and real-time RT-PCR, we characterized the root transcriptomic reprogramming of *C. hybridum* with respect to different beneficial fungi and identified specific changes in relative abundance for transcripts of genes in OM symbiosis. Genes involved in cell wall modification, reactive oxygen species detoxification, defense-related phytohormone and phosphate transport were co- induced in all the symbiotic interactions. The transcripts increased specifically by OM were related to signaling (LysM domain receptor-like kinase 3-like), cell wall degradation or reinforcement, protein metabolism and processing, defense (chitinase and mannose-specific lectin) and N, Fe transport. Among these DEGs, genes potentially functioned in C, N, P and Fe transport, hormone metabolism and signaling are of particular interest. Aside from these orchid transcripts, we also identified some putative fungal genes in symbiotic roots which were associated with plant cell wall degradation, remodeling the fungal cell wall and nutrient transport. This comprehensive dataset provides a basis for future research in exploring the molecular mechanisms underlying OM formation and functionality.

## Electronic supplementary material

Additional file 1: Table S1: Identities of the selected reference assemblies with Sanger-sequencing results of corresponding touch-down PCR products. (DOCX 15 KB)

Additional file 2: Table S2: Gene-specific primers used for qRT-PCR. (DOCX 16 KB)

Additional file 3: Figure S1: Typical co-cultivation condition of *Cymbidium hybridum* plantlets with different fungi at 15 dpi. Mock-inoculated control **(A)** and inoculated with isolate Ml01 **(B)**, ZH3A-3 **(C)** and ML01 + ZH3A-3 **(D)**. (JPEG 4 MB)

Additional file 4: Figure S2: Root staining results of each treatment at different symbiotic time points. **(A)** Roots inoculated with *E. repens* isolate ML01 at 6 days post-inoculation (dpi); **(B)** Roots inoculated with *U. nana* isolate ZH3A-3 at 6 dpi; **(C)** Roots inoculated with isolates ML01 and ZH3A-3 at 6 dpi; **(D)** Mock-inoculated roots at 6 dpi; **(E)** Roots inoculated with *E. repens* isolate ML01 at 10 dpi; **(F)** Roots inoculated with *U. nana* isolate ZH3A-3 at 10 dpi; **(G)** Roots inoculated with isolates ML01 and ZH3A-3 at 10 dpi; **(H)** Mock-inoculated roots at 10 dpi; **(I)** Roots inoculated with *E. repens* isolate ML01 at 15 dpi; **(J)** Roots inoculated with *U. nana* isolate ZH3A-3 at 15 dpi; **(K)** Roots inoculated with isolates ML01 and ZH3A-3 at 15 dpi; **(L)** Mock-inoculated roots at 15 dpi; **(M)** Roots inoculated with *E. repens* isolate ML01 at 30 dpi; **(N)** Roots inoculated with *U. nana* isolate ZH3A-3 at 30 dpi; **(O)** Roots inoculated with isolates ML01 and ZH3A-3 at 30 dpi; **(P)** Mock-inoculated roots at 30 dpi. (PNG 966 KB)

Additional file 5: ***Cymbidium hybridum*** **root transcriptome assembly- Illmina 90 bp reads.** (ZIP 6 MB)

Additional file 6: Table S3: Highly abundant transcripts in CyTr-unigenes (Top 50). (XLS 54 KB)

Additional file 7: Table S4: Pathway annotation of CyTr-unigenes. (DOC 202 KB)

Additional file 8: Table S5: Statistics of DGE sequencing. (XLSX 10 KB)

Additional file 9: Table S6: Statistics of uniquely mapped reads for each sample. (XLSX 10 KB)

Additional file 10: Table S7-S9: Lists of differentially expressed genes in different *C. hybridum*- beneficial fungi interactions. (ZIP 486 KB)

Additional file 11: Table S10: Top 100 abundant genes in symbiotic roots with positive fold change in response to symbiotic fungi. **Table S11.** Top 100 abundant genes in non-symbiotic roots with negative fold change in response to symbiotic fungi. (ZIP 144 KB)

Additional file 12: Figure S3: Hierarchical clustering of DEGs from *C. hybridum* expressed in the roots in response to isolates ML01 (CyEX21), ZH3A-3 (CyEX22), ML01 + ZH3A-3 (CyEX23)or mock inoculation (CyEX20). (PNG 25 KB)

Additional file 13: **Lists of DEGS in 20 sub-clusters based on k-means.** (ZIP 91 KB)

Additional file 14: Figure S4-S6: GO enrichment bar graphs of DEGs in the interactions between *C. hybridum* and ML01 (S4), ZH3A-3 (S5) or ML01 + ZH3A-3 (S6). (ZIP 24 KB)

Additional file 15: Table S12-S14: Lists of enriched pathways in different *C. hybridum*- beneficial fungi interactions. (ZIP 28 KB)

Additional file 16: Figure S7-S9: Scatterplot for top 20 enriched KEGG pathways of DEGs in the interactions between *C. hybridum* and ML01 (S7), ZH3A-3 (S8) or ML01 + ZH3A-3 (S9). (ZIP 22 KB)

Additional file 17: Figure S10: Comparison of expression profiles of random selected 26 genes by RNA-seq and qRT-PCR. (ZIP 158 KB)

Additional file 18: Table S15: Expression data for putative genes from *E. repens* isolate ML01 in symbiotic roots. **Table S16.** Expression data for putative genes from *U. nana* isolate ZH3A-3 in symbiotic roots. (ZIP 68 KB)

Additional file 19: Figure S11: Phylogenetic tree for the amino acid sequences of symbiosis- associated phosphate transporters from plant and fungi.The dendrogram was generated by Mega 4.0 software using ClustlW for the alignment and the neighbor-joining method for the construction of phylogeny. The plant Pi-transporters accession numbers could be obtained in [49]. Fungal phosphate transporters accession numbers: *Glomus versiforme*: GvePT, AAC49132.1; GvePT2, gi|74654973; *Rhizophagus intraradices*: RinPT, AAL37552.1; Yeast: YEPHO84, gi|1346710; *Sesbania rostrata*: SerPT, CAC28219.1; *Piriformospora indica*: PiPT1, ABI93950.1; PiPT2, CCA74169.1; PiPT3, CCA74178.1; PiPT4, CCA76379.1. The nucleotide sequences of putative phosphate transporters in our study were available in Additional file 6: Table S3. (PDF 90 KB)

## Abbreviations

ABC: ATP-binding cassette
AMs: Arbuscular mycorrhizas
AP2/ERF: APETALA2/ET response factor
ARF: Auxin response factor
bHLH: Basic helix-loop-helix
CAT5: Cationic amino acid transporter 5
CDPK: Calcium-dependent protein kinase
CKX: Cytokinin oxidase/dehydrogenase
DEGs: Differentially expressed genes
DGE: Digital Gene Expression
ECMs: Ectomycorrhizas
ETR: Ethylene receptor homolog
LHT1: Lysine histidine transporter 1
LysM-RLK: LysM-domain containing receptor-like kinases
MAF: Mycorrhizal- associated fungi
MD: Mycorrhizal dependency
MH: Heterotrophy
MX: Mixotrophs
NAATAA: Nicotianamine aminotransferase A
NGS: Next generation sequencing
NSP2: Nodulation-signaling pathway 2 protein
OM: Orchid mycorrhizae
OMF: Orchid mycorrhizal fungi
OPT: Oligopeptide transporter family
PAT: Polar auxin transport
PDA: Potato-dextrose agar
PDCD: Programmed cell death protein 4
PT: Phosphate transporter
PTR: Peptide transporter
PRG: Percentage of root growth
QC: Quiescent center
RAM1: Protein required for arbuscular mycorrhization 1
RMBT: Reads that could be mapped back to transcripts
RPKM: Reads per kilo base per million reads
SCR: SCARECROW
SPA: Suppressor of phyA-105
TFs: Transcription factors
TMT2: Tonoplast monosaccharide transporter 2
VAMP7B: Vesicle-associated membrane protein 7B
XET: Xyloglucan endotransglucosylase.
