# Supplementary material for: Deep sequencing–based comparative transcriptional profiles of Cymbidium hybridum roots in response to mycorrhizal and non-mycorrhizal beneficial fungi
Source: BMC Genomics. 2014 Aug 31;15(1):747. doi: 10.1186/1471-2164-15-747 (PMC4162972; doi:10.1186/1471-2164-15-747)
Supplement: Supplementary file 1 — Additional file 1: Table S1: Identities of the selected reference assemblies with Sanger-sequencing results of corresponding touch-down PCR products. (DOCX 15 KB) [file 12864_2014_6428_MOESM1_ESM.docx]

Table S1 Identities of the selected reference assemblies with Sanger-sequencing results of corresponding touch-down PCR products

| Assemblies ID | Foward primer(5'-3') | Reverse primer(5'-3') | PCR ID | PCR products (bp) | Identities |
| --- | --- | --- | --- | --- | --- |
| Unigene13573_All | GCTGGAGAGAGTGGCA | TGTCATAATGGCATAATCA | B1 | 716 | 99. 02% |
| Unigene2569_All | GGCTACCCAAAAGTCCAGT | GCCCTAAATCCTTCCCAG | B2 | 1430 | 96.02% |
| Unigene14999_All | TCTACAAGGGCAGAGCAC | GCAGAAAGAGAAGGCGTT | B3 | 641 | 99.84% |
| Unigene16297_All | GCGGCGTTTTTCATCG | GGCATTTCTACTCGGGGT | B4 | 1044 | 99.27% |
| Unigene10727_All | TCCTCGGAAATACACCTACA | CCACCAACTGACCCTGAT | B5 | 1100 | 100% |
| Unigene8021_All | TCACAAGGCGATGAAGGC | TTTGATGGGCAATCCGTC | B6 | 751 | 99.59% |
| Unigene15958_All | CGCATCCGTCCTCTTCAT | AAAACTAACTCCATCCCACAA | B7 | 731 | 99.86% |
| Unigene4429_All | CATACCTGTTCCGCATCG | TTCCAGCCTCCACTCCAT | B8 | 1215 | 92.25% |
| Unigene11039_All | CTCTCTCAGCCCCCGAAG | GCCACGCAACCCATACAC | B9 | 1003 | 99.44% |
| Unigene9038_All | GGTTCAGGCTGTAATGTCC | CAAAGGGTGGCTAATAAATC | B10 | 1210 | 99.37% |
| Unigene13718_All | CGTGGTGGCTTCTGGA | TGTAAGGGGTGAAATGGC | B11 | 683 | 99.55% |
| Unigene1486_All | AACACCTTACTACTATGAGCC | GAGACTATGTCCCAACCTG | B12 | 1285 | 99.32% |
| Unigene15779_All | TGGTGGAACGGTAGTGGA | TCAAAGGCTTCAAAATCATCT | B13 | 1008 | 98.49% |
| Unigene2376_All | GTTGGCTATGCTCTACCTAC | ATGCTCCTGGCTCACA | B14 | 1113 | 98.13% |
| Unigene2386_All | AGGCAACTACAACTACCA | ATTTCCAACTCCACCC | B15 | 845 | 97.99% |
| Unigene5052_All | AGGAGAAAGTGGAACAAGA | TCAAGCAATAGAGGGACC | B16 | 582 | 99.39% |
| Unigene13170_All | ACCGTTGAGCGGCATT | TGGTGAAAAGTAAAGAGCGA | B17 | 1281 | 99.49% |
| Unigene13279_All | CTCCTTATTCCCGTCCAG | CTTGCCACTCTATTGTCCAC | B18 | 1419 | 99.32% |
