# Supplementary material for: Deep sequencing–based comparative transcriptional profiles of Cymbidium hybridum roots in response to mycorrhizal and non-mycorrhizal beneficial fungi
Source: BMC Genomics. 2014 Aug 31;15(1):747. doi: 10.1186/1471-2164-15-747 (PMC4162972; doi:10.1186/1471-2164-15-747)
Supplement: Supplementary file 2 — Additional file 2: Table S2: Gene-specific primers used for qRT-PCR. (DOCX 16 KB) [file 12864_2014_6428_MOESM2_ESM.docx]

| Unigene ID | PCR ID | Forward primer(5'-3') | Reverse primer(5'-3') | PCR products (bp) |
| --- | --- | --- | --- | --- |
| Unigene1724_All | D5 | GGCGTTCAGGTTGATAATG | TTTTGGGACTCACTACAGGA | 152 |
| Unigene1869_All | D6 | GTTCGCATTCGGAGTAGTC | CCGTCATCTCGCTTATTTC | 269 |
| Unigene2625_All | D9 | CGACCACGGAGCGAACT | TCCCAACCCTGGCAACT | 232 |
| Unigene4130_All | D11 | GTTTGACCAATAGCGAGC | GCAGAAAAGCCAACACAG | 154 |
| Unigene4239_All | D12 | TTGGTGCCCTTGTTCCTG | CGCTGCCTTATGCTTTCC | 289 |
| Unigene6086_All | D13 | GGCGACAAGATGGGACG | TGATGGTTGCGGATAGGG | 181 |
| Unigene8895_All | D18 | TTTGATAGCCATTTCTTCTG | TGAGTCGGTCCTTGTGAT | 246 |
| Unigene10786_All | D23 | GCCATTCTTTCTCGCTTC | CCTCTGGTCCCTTGTATCC | 163 |
| Unigene12197_All | D29 | AAGAAGAGCAGGGTATGGC | CTATCGGCGGGAAGGTA | 199 |
| Unigene13078_All | D31 | AATGTATCCTTCCGTGGG | CATAGAGAGATTCAGGCGTC | 137 |
| Unigene13219_All | D33 | TCCAGTTCGTCGCCCTC | CCAAGCAAAGTAGCCGTTC | 255 |
| Unigene13639_All | D36 | CTGAACCCACAAGATAACCT | CTGGAGCGTGTCACAAATA | 113 |
| Unigene14415_All | D38 | AAATGGCTGGGATTCGTG | GATGCGGTCGGAGGCTA | 189 |
| Unigene15955_All | D42 | ATTGCTCCCGTCTATACTGC | GGAATCGGATGTCTTTGCT | 291 |
| Unigene13573_All | B1 | CCTGGCGAGCATCTGGTT | AGACGGCGAATAGGTGGG | 152 |
| Unigene2569_All | B2 | GAACTGATTATTGCGTCCG | TATGAGCAGCACAAAGGC | 210 |
| Unigene14999_All | B3 | GAAGGATGGGTTGTCTGC | CGCTTTCGCTGTTTGAGT | 130 |
| Unigene16297_All | B4 | CTGCCATCACAACATTCG | CATTCAGGCTTACTGGTCTT | 133 |
| Unigene10727_All | B5 | GAATCTGGCAGGGAGCAT | ACGGGAAGCAACGGAGT | 208 |
| Unigene8021_All | B6 | CCACGGCGAGCGTTCTTA | TCGGCATCCTCTTTGTATCAGC | 162 |
| Unigene15958_All | B7 | GGTCCTGCTGGTCAAGAA | ATGGCTCGGATGGTGG | 155 |
| Unigene4429_All | B8 | AAGATTGTATCGCCTCGC | TGTTGGGGTCATAGTTTCC | 146 |
| Unigene11039_All | B9 | GGTATCAACCGCTTCGTC | AGTTTTTGGAGGGAGTCG | 119 |
| Unigene9038_All | B10 | CCGAGTGGGGTAATGGA | GAGGAGGTCAGAGTAGCAGG | 140 |
| Unigene13718_All | B11 | GGAGGAGCAGGATTATGGG | GGAGTTGAGGCGATGGAA | 283 |
| Unigene1486_All | B12 | GACTTGCGAGAGAACTGACG | GGCTACCTCCACCCTGC | 218 |
| Unigene15779_All | B13 | AGTGGTGTTGCTGCTCTG | ATCAAGTATGGCATCGTAT | 216 |
| Unigene2376_All | B14 | CTGAAGAAACAAAGCAGATG | AAAAAGCGGTCGTAGAGG | 214 |
| Unigene2386_All | B15 | GGGCTTGTGGGACCTAAA | GTGTGTGCTGGTGGGATG | 194 |
| Unigene5052_All | B16 | GCCTCTTCCATCCTCCTA | GCTCAAACACTCATCCGA | 171 |
| Unigene13170_All | B17 | ACGCCAGACGCCTATG | TGGTATGCCGTGGTTGAT | 153 |
| Unigene13279_All | B18 | AAAAGAGCACAGAGAGTTG | CTATTGTCCACCCACCAC | 173 |
| Unigene11515_All | B21 | TTTTCAAGGAGAAGAGGGTA | ACAGAGTTGGGATTAGGCT | 144 |
| Unigene1346_All | B24 | GCCCTTGTATTGCTGTTGTC | TTTCCAGTGATTCCACGC | 178 |

Table S2 Gene-specific primers used for qRT-PCR
