# Supplementary figures and images for: Deep sequencing–based comparative transcriptional profiles of Cymbidium hybridum roots in response to mycorrhizal and non-mycorrhizal beneficial fungi
Source: BMC Genomics. 2014 Aug 31;15(1):747. doi: 10.1186/1471-2164-15-747 (PMC4162972; doi:10.1186/1471-2164-15-747)

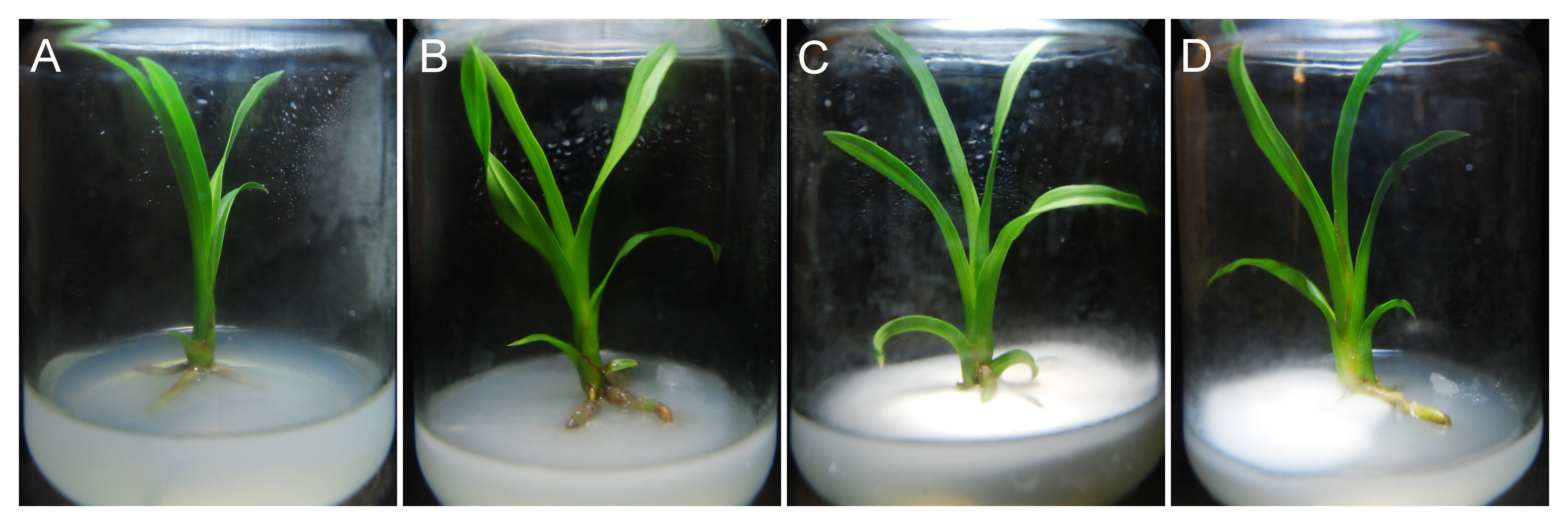

Supplement: Supplementary file 3 — Additional file 3: Figure S1: Typical co-cultivation condition of Cymbidium hybridum plantlets with different fungi at 15 dpi. Mock-inoculated control (A) and inoculated with isolate Ml01 (B), ZH3A-3 (C) and ML01 + ZH3A-3 (D). (JPEG 4 MB) [file 12864_2014_6428_MOESM3_ESM.jpeg]

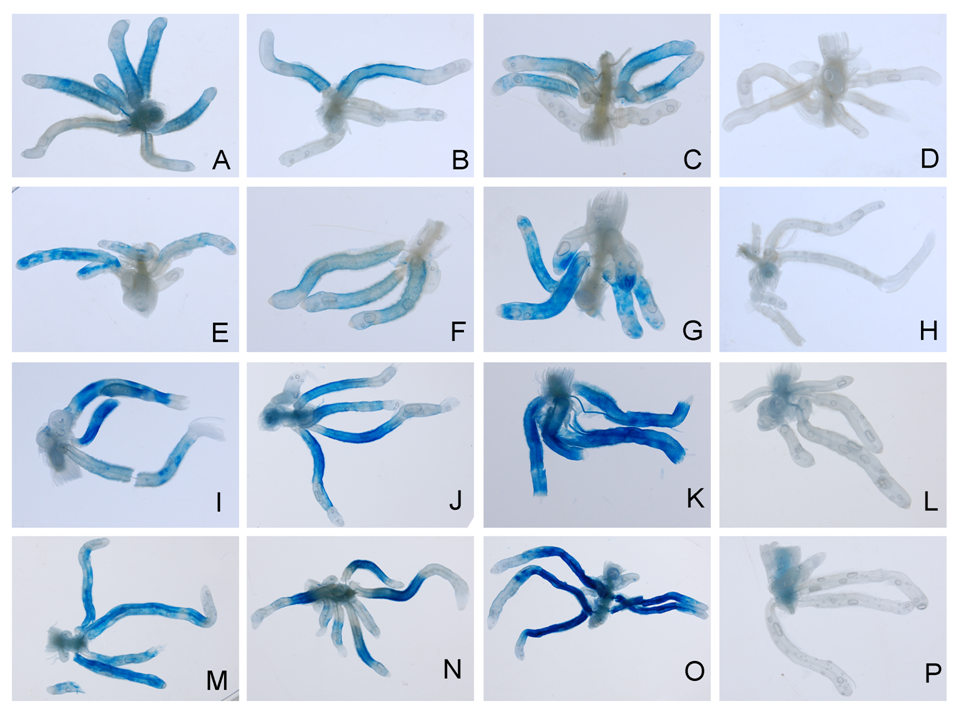

Supplement: Supplementary file 4 — Additional file 4: Figure S2: Root staining results of each treatment at different symbiotic time points. (A) Roots inoculated with E. repens isolate ML01 at 6 days post-inoculation (dpi); (B) Roots inoculated with U. nana isolate ZH3A-3 at 6 dpi; (C) Roots inoculated with isolates ML01 and ZH3A-3 at 6 dpi; (D) Mock-inoculated roots at 6 dpi; (E) Roots inoculated with E. repens isolate ML01 at 10 dpi; (F) Roots inoculated with U. nana isolate ZH3A-3 at 10 dpi; (G) Roots inoculated with isolates ML01 and ZH3A-3 at 10 dpi; (H) Mock-inoculated roots at 10 dpi; (I) Roots inoculated with E. repens isolate ML01 at 15 dpi; (J) Roots inoculated with U. nana isolate ZH3A-3 at 15 dpi; (K) Roots inoculated with isolates ML01 and ZH3A-3 at 15 dpi; (L) Mock-inoculated roots at 15 dpi; (M) Roots inoculated with E. repens isolate ML01 at 30 dpi; (N) Roots inoculated with U. nana isolate ZH3A-3 at 30 dpi; (O) Roots inoculated with isolates ML01 and ZH3A-3 at 30 dpi; (P) Mock-inoculated roots at 30 dpi. (PNG 966 KB) [file 12864_2014_6428_MOESM4_ESM.png]

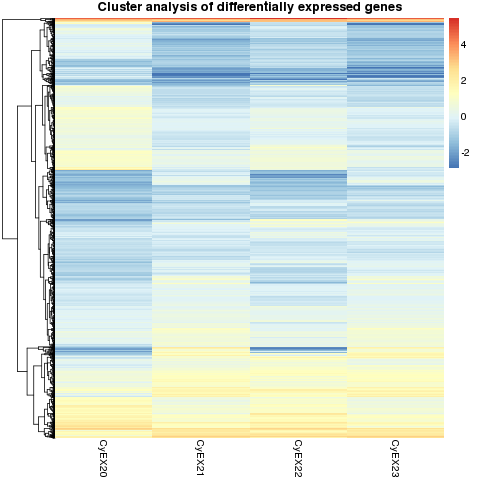

Supplement: Supplementary file 12 — Additional file 12: Figure S3: Hierarchical clustering of DEGs from C. hybridum expressed in the roots in response to isolates ML01 (CyEX21), ZH3A-3 (CyEX22), ML01 + ZH3A-3 (CyEX23)or mock inoculation (CyEX20). (PNG 25 KB) [file 12864_2014_6428_MOESM12_ESM.png]

Enriched GO Terms  
(CyEX21vsCyEX20)

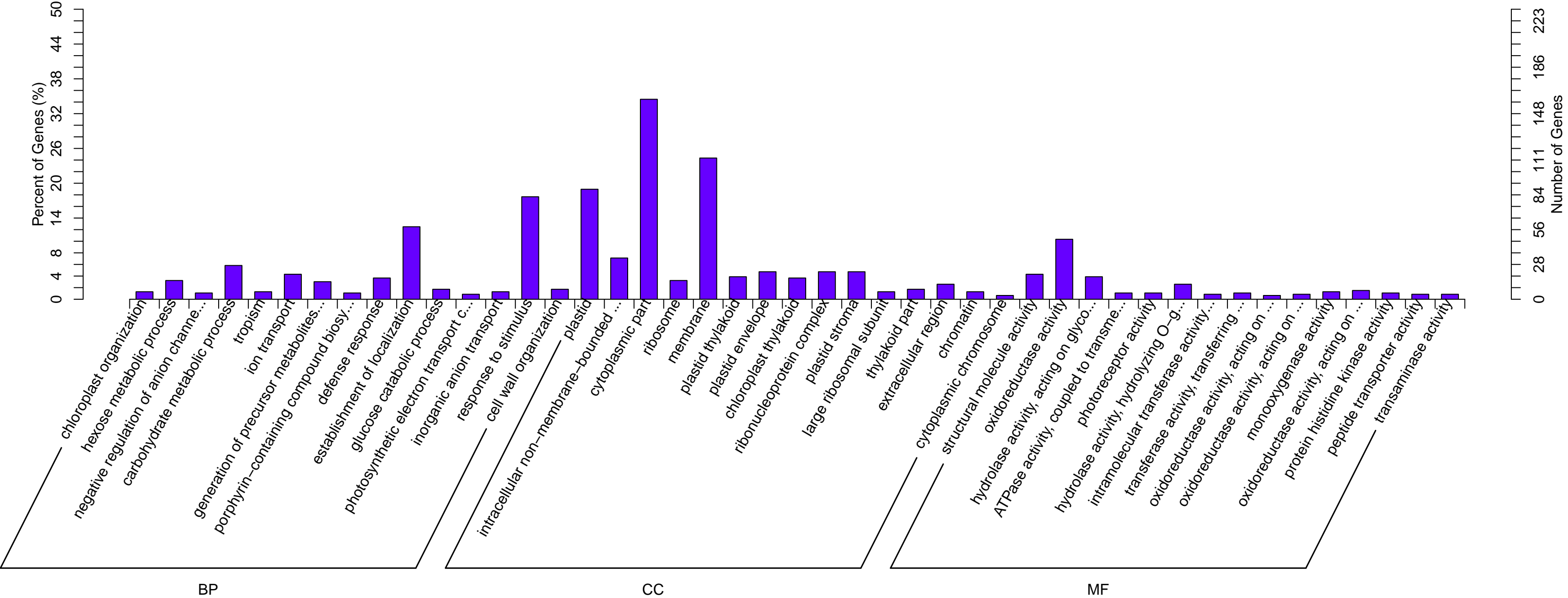

Supplement: Supplementary file 14 — Additional file 14: Figure S4-S6: GO enrichment bar graphs of DEGs in the interactions between C. hybridum and ML01 (S4), ZH3A-3 (S5) or ML01 + ZH3A-3 (S6). (ZIP 24 KB) [file 12864_2014_6428_MOESM14_ESM.zip › Additional 14/Figure S4 CyEX21vsCyEX20.pdf]

Enriched GO Terms  
(CyEX22vsCyEX20)

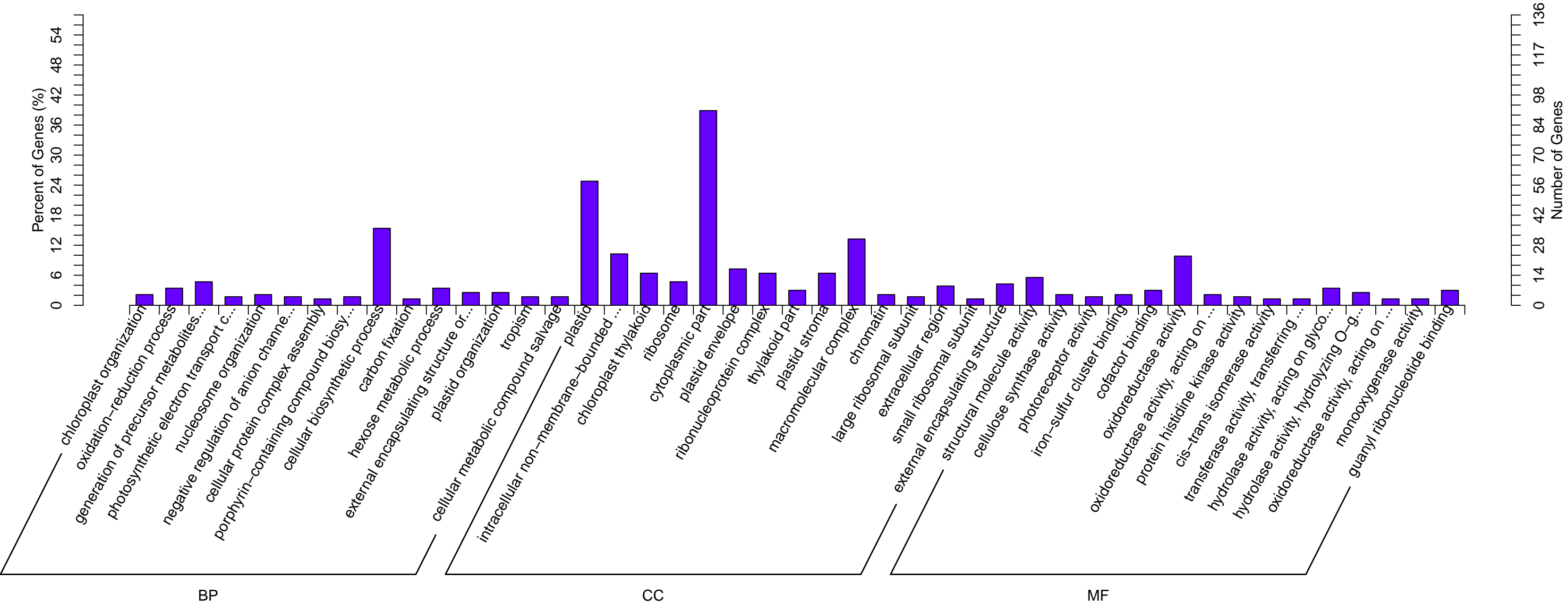

Supplement: Supplementary file 14 — Additional file 14: Figure S4-S6: GO enrichment bar graphs of DEGs in the interactions between C. hybridum and ML01 (S4), ZH3A-3 (S5) or ML01 + ZH3A-3 (S6). (ZIP 24 KB) [file 12864_2014_6428_MOESM14_ESM.zip › Additional 14/Figure S5 CyEX22vsCyEX20.pdf]

Enriched GO Terms  
(CyEX23vsCyEX20)

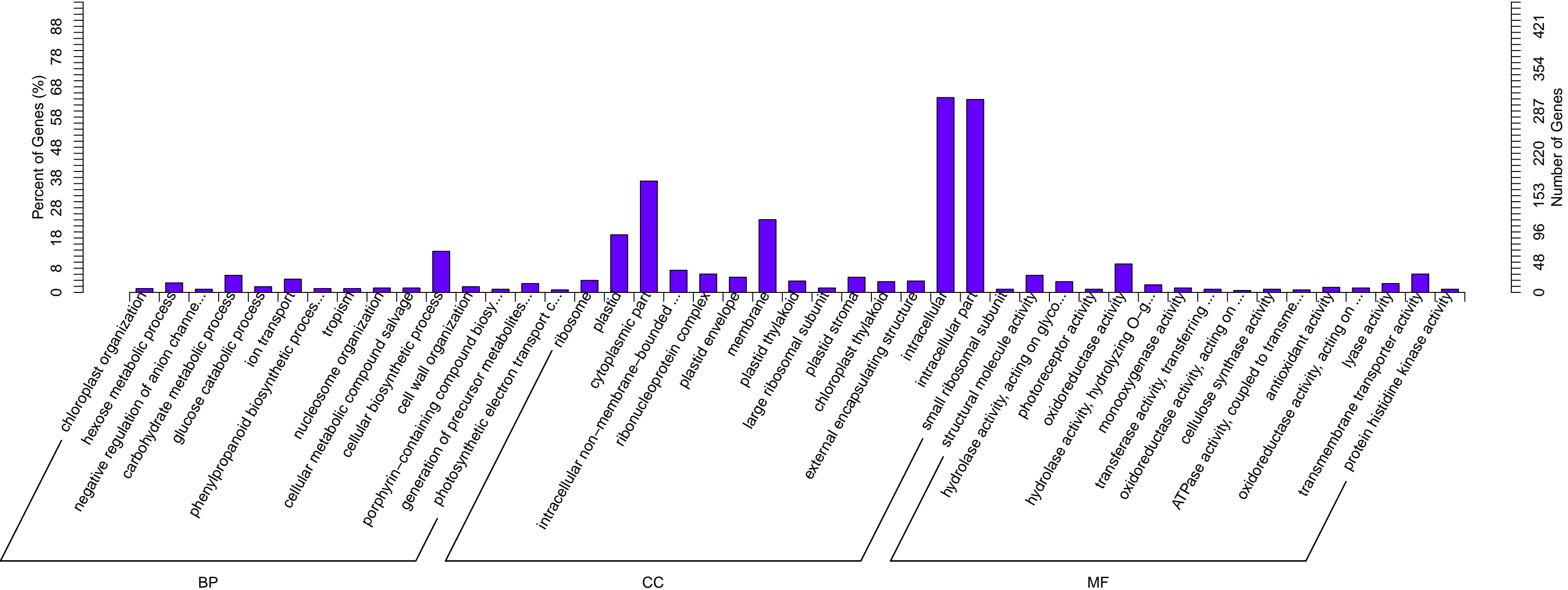

Supplement: Supplementary file 14 — Additional file 14: Figure S4-S6: GO enrichment bar graphs of DEGs in the interactions between C. hybridum and ML01 (S4), ZH3A-3 (S5) or ML01 + ZH3A-3 (S6). (ZIP 24 KB) [file 12864_2014_6428_MOESM14_ESM.zip › Additional 14/Figure S6 CyEX23vsCyEX20.pdf]

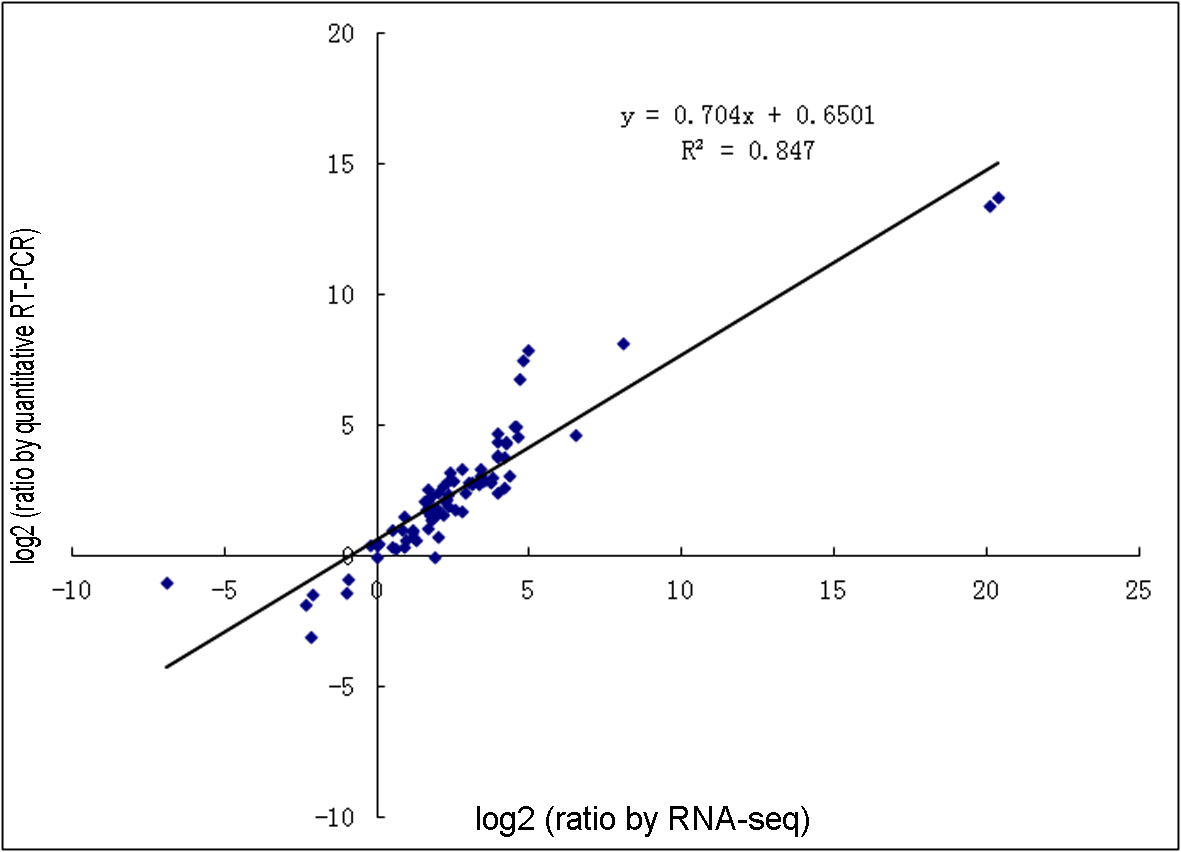

Supplement: Supplementary file 17 — Additional file 17: Figure S10: Comparison of expression profiles of random selected 26 genes by RNA-seq and qRT-PCR. (ZIP 158 KB) [file 12864_2014_6428_MOESM17_ESM.zip › Additional file 17 (Figure S10) as TIF.tif]
