# Supplementary material for: Deep sequencing–based comparative transcriptional profiles of Cymbidium hybridum roots in response to mycorrhizal and non-mycorrhizal beneficial fungi
Source: BMC Genomics. 2014 Aug 31;15(1):747. doi: 10.1186/1471-2164-15-747 (PMC4162972; doi:10.1186/1471-2164-15-747)
Supplement: Supplementary file 7 — Additional file 7: Table S4: Pathway annotation of CyTr-unigenes. (DOC 202 KB) [file 12864_2014_6428_MOESM7_ESM.doc]

Table S4 Pathway annotation of CyTr-unigenes

|  | **Pathway** | **DEGs with pathway annotation (5721)** | **Pathway ID** |
| --- | --- | --- | --- |
| 1 | [Metabolic pathways](../../../../F:%5C%E5%8D%8E%E5%A4%A7%E6%B5%8B%E5%BA%8F%5Ctranscriptome%20(trinity-Unigene-all)%5CCymbidium%5CCymbidium%5Cannotation%5CKEGG%5CCyTr-Unigene.fa.htm#gene1) | 1261 (22.04%) | ko01100 |
| 2 | [Biosynthesis of secondary metabolites](../../../../F:%5C%E5%8D%8E%E5%A4%A7%E6%B5%8B%E5%BA%8F%5Ctranscriptome%20(trinity-Unigene-all)%5CCymbidium%5CCymbidium%5Cannotation%5CKEGG%5CCyTr-Unigene.fa.htm#gene2) | 545 (9.53%) | ko01110 |
| 3 | [Spliceosome](../../../../F:%5C%E5%8D%8E%E5%A4%A7%E6%B5%8B%E5%BA%8F%5Ctranscriptome%20(trinity-Unigene-all)%5CCymbidium%5CCymbidium%5Cannotation%5CKEGG%5CCyTr-Unigene.fa.htm#gene3) | 531 (9.28%) | ko03040 |
| 4 | [Plant-pathogen interaction](../../../../F:%5C%E5%8D%8E%E5%A4%A7%E6%B5%8B%E5%BA%8F%5Ctranscriptome%20(trinity-Unigene-all)%5CCymbidium%5CCymbidium%5Cannotation%5CKEGG%5CCyTr-Unigene.fa.htm#gene4) | 439 (7.67%) | ko04626 |
| 5 | [Purine metabolism](../../../../F:%5C%E5%8D%8E%E5%A4%A7%E6%B5%8B%E5%BA%8F%5Ctranscriptome%20(trinity-Unigene-all)%5CCymbidium%5CCymbidium%5Cannotation%5CKEGG%5CCyTr-Unigene.fa.htm#gene5) | 334 (5.84%) | ko00230 |
| 6 | [Pyrimidine metabolism](../../../../F:%5C%E5%8D%8E%E5%A4%A7%E6%B5%8B%E5%BA%8F%5Ctranscriptome%20(trinity-Unigene-all)%5CCymbidium%5CCymbidium%5Cannotation%5CKEGG%5CCyTr-Unigene.fa.htm#gene6) | 319 (5.58%) | ko00240 |
| 7 | [RNA polymerase](../../../../F:%5C%E5%8D%8E%E5%A4%A7%E6%B5%8B%E5%BA%8F%5Ctranscriptome%20(trinity-Unigene-all)%5CCymbidium%5CCymbidium%5Cannotation%5CKEGG%5CCyTr-Unigene.fa.htm#gene7) | 260 (4.54%) | ko03020 |
| 8 | [Protein processing in endoplasmic reticulum](../../../../F:%5C%E5%8D%8E%E5%A4%A7%E6%B5%8B%E5%BA%8F%5Ctranscriptome%20(trinity-Unigene-all)%5CCymbidium%5CCymbidium%5Cannotation%5CKEGG%5CCyTr-Unigene.fa.htm#gene8) | 154 (2.69%) | ko04141 |
| 9 | [Ubiquitin mediated proteolysis](../../../../F:%5C%E5%8D%8E%E5%A4%A7%E6%B5%8B%E5%BA%8F%5Ctranscriptome%20(trinity-Unigene-all)%5CCymbidium%5CCymbidium%5Cannotation%5CKEGG%5CCyTr-Unigene.fa.htm#gene9) | 132 (2.31%) | ko04120 |
| 10 | [Ribosome](../../../../F:%5C%E5%8D%8E%E5%A4%A7%E6%B5%8B%E5%BA%8F%5Ctranscriptome%20(trinity-Unigene-all)%5CCymbidium%5CCymbidium%5Cannotation%5CKEGG%5CCyTr-Unigene.fa.htm#gene10) | 120 (2.1%) | ko03010 |
| 11 | [Endocytosis](../../../../F:%5C%E5%8D%8E%E5%A4%A7%E6%B5%8B%E5%BA%8F%5Ctranscriptome%20(trinity-Unigene-all)%5CCymbidium%5CCymbidium%5Cannotation%5CKEGG%5CCyTr-Unigene.fa.htm#gene11) | 109 (1.91%) | ko04144 |
| 12 | [Starch and sucrose metabolism](../../../../F:%5C%E5%8D%8E%E5%A4%A7%E6%B5%8B%E5%BA%8F%5Ctranscriptome%20(trinity-Unigene-all)%5CCymbidium%5CCymbidium%5Cannotation%5CKEGG%5CCyTr-Unigene.fa.htm#gene12) | 100 (1.75%) | ko00500 |
| 13 | [Phenylpropanoid biosynthesis](../../../../F:%5C%E5%8D%8E%E5%A4%A7%E6%B5%8B%E5%BA%8F%5Ctranscriptome%20(trinity-Unigene-all)%5CCymbidium%5CCymbidium%5Cannotation%5CKEGG%5CCyTr-Unigene.fa.htm#gene13) | 100 (1.75%) | ko00940 |
| 14 | [RNA degradation](../../../../F:%5C%E5%8D%8E%E5%A4%A7%E6%B5%8B%E5%BA%8F%5Ctranscriptome%20(trinity-Unigene-all)%5CCymbidium%5CCymbidium%5Cannotation%5CKEGG%5CCyTr-Unigene.fa.htm#gene14) | 95 (1.66%) | ko03018 |
| 15 | [Cysteine and methionine metabolism](../../../../F:%5C%E5%8D%8E%E5%A4%A7%E6%B5%8B%E5%BA%8F%5Ctranscriptome%20(trinity-Unigene-all)%5CCymbidium%5CCymbidium%5Cannotation%5CKEGG%5CCyTr-Unigene.fa.htm#gene15) | 90 (1.57%) | ko00270 |
| 16 | [Circadian rhythm - plant](../../../../F:%5C%E5%8D%8E%E5%A4%A7%E6%B5%8B%E5%BA%8F%5Ctranscriptome%20(trinity-Unigene-all)%5CCymbidium%5CCymbidium%5Cannotation%5CKEGG%5CCyTr-Unigene.fa.htm#gene16) | 81 (1.42%) | ko04712 |
| 17 | [Nucleotide excision repair](../../../../F:%5C%E5%8D%8E%E5%A4%A7%E6%B5%8B%E5%BA%8F%5Ctranscriptome%20(trinity-Unigene-all)%5CCymbidium%5CCymbidium%5Cannotation%5CKEGG%5CCyTr-Unigene.fa.htm#gene17) | 72 (1.26%) | ko03420 |
| 18 | [ABC transporters](../../../../F:%5C%E5%8D%8E%E5%A4%A7%E6%B5%8B%E5%BA%8F%5Ctranscriptome%20(trinity-Unigene-all)%5CCymbidium%5CCymbidium%5Cannotation%5CKEGG%5CCyTr-Unigene.fa.htm#gene18) | 70 (1.22%) | ko02010 |
| 19 | [Glycerophospholipid metabolism](../../../../F:%5C%E5%8D%8E%E5%A4%A7%E6%B5%8B%E5%BA%8F%5Ctranscriptome%20(trinity-Unigene-all)%5CCymbidium%5CCymbidium%5Cannotation%5CKEGG%5CCyTr-Unigene.fa.htm#gene19) | 70 (1.22%) | ko00564 |
| 20 | [Oxidative phosphorylation](../../../../F:%5C%E5%8D%8E%E5%A4%A7%E6%B5%8B%E5%BA%8F%5Ctranscriptome%20(trinity-Unigene-all)%5CCymbidium%5CCymbidium%5Cannotation%5CKEGG%5CCyTr-Unigene.fa.htm#gene20) | 67 (1.17%) | ko00190 |
| 21 | [Basal transcription factors](../../../../F:%5C%E5%8D%8E%E5%A4%A7%E6%B5%8B%E5%BA%8F%5Ctranscriptome%20(trinity-Unigene-all)%5CCymbidium%5CCymbidium%5Cannotation%5CKEGG%5CCyTr-Unigene.fa.htm#gene21) | 66 (1.15%) | ko03022 |
| 22 | [Peroxisome](../../../../F:%5C%E5%8D%8E%E5%A4%A7%E6%B5%8B%E5%BA%8F%5Ctranscriptome%20(trinity-Unigene-all)%5CCymbidium%5CCymbidium%5Cannotation%5CKEGG%5CCyTr-Unigene.fa.htm#gene22) | 65 (1.14%) | ko04146 |
| 23 | [Phagosome](../../../../F:%5C%E5%8D%8E%E5%A4%A7%E6%B5%8B%E5%BA%8F%5Ctranscriptome%20(trinity-Unigene-all)%5CCymbidium%5CCymbidium%5Cannotation%5CKEGG%5CCyTr-Unigene.fa.htm#gene23) | 65 (1.14%) | ko04145 |
| 24 | [Amino sugar and nucleotide sugar metabolism](../../../../F:%5C%E5%8D%8E%E5%A4%A7%E6%B5%8B%E5%BA%8F%5Ctranscriptome%20(trinity-Unigene-all)%5CCymbidium%5CCymbidium%5Cannotation%5CKEGG%5CCyTr-Unigene.fa.htm#gene24) | 61 (1.07%) | ko00520 |
| 25 | [Glycolysis / Gluconeogenesis](../../../../F:%5C%E5%8D%8E%E5%A4%A7%E6%B5%8B%E5%BA%8F%5Ctranscriptome%20(trinity-Unigene-all)%5CCymbidium%5CCymbidium%5Cannotation%5CKEGG%5CCyTr-Unigene.fa.htm#gene25) | 57 (1%) | ko00010 |
| 26 | [Phenylalanine metabolism](../../../../F:%5C%E5%8D%8E%E5%A4%A7%E6%B5%8B%E5%BA%8F%5Ctranscriptome%20(trinity-Unigene-all)%5CCymbidium%5CCymbidium%5Cannotation%5CKEGG%5CCyTr-Unigene.fa.htm#gene26) | 56 (0.98%) | ko00360 |
| 27 | [Homologous recombination](../../../../F:%5C%E5%8D%8E%E5%A4%A7%E6%B5%8B%E5%BA%8F%5Ctranscriptome%20(trinity-Unigene-all)%5CCymbidium%5CCymbidium%5Cannotation%5CKEGG%5CCyTr-Unigene.fa.htm#gene27) | 55 (0.96%) | ko03440 |
| 28 | [Limonene and pinene degradation](../../../../F:%5C%E5%8D%8E%E5%A4%A7%E6%B5%8B%E5%BA%8F%5Ctranscriptome%20(trinity-Unigene-all)%5CCymbidium%5CCymbidium%5Cannotation%5CKEGG%5CCyTr-Unigene.fa.htm#gene28) | 55 (0.96%) | ko00903 |
| 29 | [Nitrogen metabolism](../../../../F:%5C%E5%8D%8E%E5%A4%A7%E6%B5%8B%E5%BA%8F%5Ctranscriptome%20(trinity-Unigene-all)%5CCymbidium%5CCymbidium%5Cannotation%5CKEGG%5CCyTr-Unigene.fa.htm#gene29) | 55 (0.96%) | ko00910 |
| 30 | [DNA replication](../../../../F:%5C%E5%8D%8E%E5%A4%A7%E6%B5%8B%E5%BA%8F%5Ctranscriptome%20(trinity-Unigene-all)%5CCymbidium%5CCymbidium%5Cannotation%5CKEGG%5CCyTr-Unigene.fa.htm#gene30) | 54 (0.94%) | ko03030 |
| 31 | [Stilbenoid, diarylheptanoid and gingerol biosynthesis](../../../../F:%5C%E5%8D%8E%E5%A4%A7%E6%B5%8B%E5%BA%8F%5Ctranscriptome%20(trinity-Unigene-all)%5CCymbidium%5CCymbidium%5Cannotation%5CKEGG%5CCyTr-Unigene.fa.htm#gene31) | 54 (0.94%) | ko00945 |
| 32 | [Pyruvate metabolism](../../../../F:%5C%E5%8D%8E%E5%A4%A7%E6%B5%8B%E5%BA%8F%5Ctranscriptome%20(trinity-Unigene-all)%5CCymbidium%5CCymbidium%5Cannotation%5CKEGG%5CCyTr-Unigene.fa.htm#gene32) | 52 (0.91%) | ko00620 |
| 33 | [Zeatin biosynthesis](../../../../F:%5C%E5%8D%8E%E5%A4%A7%E6%B5%8B%E5%BA%8F%5Ctranscriptome%20(trinity-Unigene-all)%5CCymbidium%5CCymbidium%5Cannotation%5CKEGG%5CCyTr-Unigene.fa.htm#gene33) | 50 (0.87%) | ko00908 |
| 34 | [Flavonoid biosynthesis](../../../../F:%5C%E5%8D%8E%E5%A4%A7%E6%B5%8B%E5%BA%8F%5Ctranscriptome%20(trinity-Unigene-all)%5CCymbidium%5CCymbidium%5Cannotation%5CKEGG%5CCyTr-Unigene.fa.htm#gene34) | 47 (0.82%) | ko00941 |
| 35 | [Glutathione metabolism](../../../../F:%5C%E5%8D%8E%E5%A4%A7%E6%B5%8B%E5%BA%8F%5Ctranscriptome%20(trinity-Unigene-all)%5CCymbidium%5CCymbidium%5Cannotation%5CKEGG%5CCyTr-Unigene.fa.htm#gene35) | 45 (0.79%) | ko00480 |
| 36 | [Phosphatidylinositol signaling system](../../../../F:%5C%E5%8D%8E%E5%A4%A7%E6%B5%8B%E5%BA%8F%5Ctranscriptome%20(trinity-Unigene-all)%5CCymbidium%5CCymbidium%5Cannotation%5CKEGG%5CCyTr-Unigene.fa.htm#gene36) | 45 (0.79%) | ko04070 |
| 37 | [Aminoacyl-tRNA biosynthesis](../../../../F:%5C%E5%8D%8E%E5%A4%A7%E6%B5%8B%E5%BA%8F%5Ctranscriptome%20(trinity-Unigene-all)%5CCymbidium%5CCymbidium%5Cannotation%5CKEGG%5CCyTr-Unigene.fa.htm#gene37) | 42 (0.73%) | ko00970 |
| 38 | [Galactose metabolism](../../../../F:%5C%E5%8D%8E%E5%A4%A7%E6%B5%8B%E5%BA%8F%5Ctranscriptome%20(trinity-Unigene-all)%5CCymbidium%5CCymbidium%5Cannotation%5CKEGG%5CCyTr-Unigene.fa.htm#gene38) | 41 (0.72%) | ko00052 |
| 39 | [Lysine degradation](../../../../F:%5C%E5%8D%8E%E5%A4%A7%E6%B5%8B%E5%BA%8F%5Ctranscriptome%20(trinity-Unigene-all)%5CCymbidium%5CCymbidium%5Cannotation%5CKEGG%5CCyTr-Unigene.fa.htm#gene39) | 40 (0.7%) | ko00310 |
| 40 | [Arginine and proline metabolism](../../../../F:%5C%E5%8D%8E%E5%A4%A7%E6%B5%8B%E5%BA%8F%5Ctranscriptome%20(trinity-Unigene-all)%5CCymbidium%5CCymbidium%5Cannotation%5CKEGG%5CCyTr-Unigene.fa.htm#gene40) | 40 (0.7%) | ko00330 |
| 41 | [Glycerolipid metabolism](../../../../F:%5C%E5%8D%8E%E5%A4%A7%E6%B5%8B%E5%BA%8F%5Ctranscriptome%20(trinity-Unigene-all)%5CCymbidium%5CCymbidium%5Cannotation%5CKEGG%5CCyTr-Unigene.fa.htm#gene41) | 40 (0.7%) | ko00561 |
| 42 | [Ascorbate and aldarate metabolism](../../../../F:%5C%E5%8D%8E%E5%A4%A7%E6%B5%8B%E5%BA%8F%5Ctranscriptome%20(trinity-Unigene-all)%5CCymbidium%5CCymbidium%5Cannotation%5CKEGG%5CCyTr-Unigene.fa.htm#gene42) | 38 (0.66%) | ko00053 |
| 43 | [Carbon fixation in photosynthetic organisms](../../../../F:%5C%E5%8D%8E%E5%A4%A7%E6%B5%8B%E5%BA%8F%5Ctranscriptome%20(trinity-Unigene-all)%5CCymbidium%5CCymbidium%5Cannotation%5CKEGG%5CCyTr-Unigene.fa.htm#gene43) | 38 (0.66%) | ko00710 |
| 44 | [Base excision repair](../../../../F:%5C%E5%8D%8E%E5%A4%A7%E6%B5%8B%E5%BA%8F%5Ctranscriptome%20(trinity-Unigene-all)%5CCymbidium%5CCymbidium%5Cannotation%5CKEGG%5CCyTr-Unigene.fa.htm#gene44) | 38 (0.66%) | ko03410 |
| 45 | [Mismatch repair](../../../../F:%5C%E5%8D%8E%E5%A4%A7%E6%B5%8B%E5%BA%8F%5Ctranscriptome%20(trinity-Unigene-all)%5CCymbidium%5CCymbidium%5Cannotation%5CKEGG%5CCyTr-Unigene.fa.htm#gene45) | 37 (0.65%) | ko03430 |
| 46 | [alpha-Linolenic acid metabolism](../../../../F:%5C%E5%8D%8E%E5%A4%A7%E6%B5%8B%E5%BA%8F%5Ctranscriptome%20(trinity-Unigene-all)%5CCymbidium%5CCymbidium%5Cannotation%5CKEGG%5CCyTr-Unigene.fa.htm#gene46) | 37 (0.65%) | ko00592 |
| 47 | [Protein export](../../../../F:%5C%E5%8D%8E%E5%A4%A7%E6%B5%8B%E5%BA%8F%5Ctranscriptome%20(trinity-Unigene-all)%5CCymbidium%5CCymbidium%5Cannotation%5CKEGG%5CCyTr-Unigene.fa.htm#gene47) | 36 (0.63%) | ko03060 |
| 48 | [Cyanoamino acid metabolism](../../../../F:%5C%E5%8D%8E%E5%A4%A7%E6%B5%8B%E5%BA%8F%5Ctranscriptome%20(trinity-Unigene-all)%5CCymbidium%5CCymbidium%5Cannotation%5CKEGG%5CCyTr-Unigene.fa.htm#gene48) | 36 (0.63%) | ko00460 |
| 49 | [Butanoate metabolism](../../../../F:%5C%E5%8D%8E%E5%A4%A7%E6%B5%8B%E5%BA%8F%5Ctranscriptome%20(trinity-Unigene-all)%5CCymbidium%5CCymbidium%5Cannotation%5CKEGG%5CCyTr-Unigene.fa.htm#gene49) | 35 (0.61%) | ko00650 |
| 50 | [Alanine, aspartate and glutamate metabolism](../../../../F:%5C%E5%8D%8E%E5%A4%A7%E6%B5%8B%E5%BA%8F%5Ctranscriptome%20(trinity-Unigene-all)%5CCymbidium%5CCymbidium%5Cannotation%5CKEGG%5CCyTr-Unigene.fa.htm#gene50) | 35 (0.61%) | ko00250 |
| 51 | [Fructose and mannose metabolism](../../../../F:%5C%E5%8D%8E%E5%A4%A7%E6%B5%8B%E5%BA%8F%5Ctranscriptome%20(trinity-Unigene-all)%5CCymbidium%5CCymbidium%5Cannotation%5CKEGG%5CCyTr-Unigene.fa.htm#gene51) | 35 (0.61%) | ko00051 |
| 52 | [Glycine, serine and threonine metabolism](../../../../F:%5C%E5%8D%8E%E5%A4%A7%E6%B5%8B%E5%BA%8F%5Ctranscriptome%20(trinity-Unigene-all)%5CCymbidium%5CCymbidium%5Cannotation%5CKEGG%5CCyTr-Unigene.fa.htm#gene52) | 34 (0.59%) | ko00260 |
| 53 | [Linoleic acid metabolism](../../../../F:%5C%E5%8D%8E%E5%A4%A7%E6%B5%8B%E5%BA%8F%5Ctranscriptome%20(trinity-Unigene-all)%5CCymbidium%5CCymbidium%5Cannotation%5CKEGG%5CCyTr-Unigene.fa.htm#gene53) | 33 (0.58%) | ko00591 |
| 54 | [Porphyrin and chlorophyll metabolism](../../../../F:%5C%E5%8D%8E%E5%A4%A7%E6%B5%8B%E5%BA%8F%5Ctranscriptome%20(trinity-Unigene-all)%5CCymbidium%5CCymbidium%5Cannotation%5CKEGG%5CCyTr-Unigene.fa.htm#gene54) | 33 (0.58%) | ko00860 |
| 55 | [Ether lipid metabolism](../../../../F:%5C%E5%8D%8E%E5%A4%A7%E6%B5%8B%E5%BA%8F%5Ctranscriptome%20(trinity-Unigene-all)%5CCymbidium%5CCymbidium%5Cannotation%5CKEGG%5CCyTr-Unigene.fa.htm#gene55) | 33 (0.58%) | ko00565 |
| 56 | [Pentose phosphate pathway](../../../../F:%5C%E5%8D%8E%E5%A4%A7%E6%B5%8B%E5%BA%8F%5Ctranscriptome%20(trinity-Unigene-all)%5CCymbidium%5CCymbidium%5Cannotation%5CKEGG%5CCyTr-Unigene.fa.htm#gene56) | 31 (0.54%) | ko00030 |
| 57 | [Inositol phosphate metabolism](../../../../F:%5C%E5%8D%8E%E5%A4%A7%E6%B5%8B%E5%BA%8F%5Ctranscriptome%20(trinity-Unigene-all)%5CCymbidium%5CCymbidium%5Cannotation%5CKEGG%5CCyTr-Unigene.fa.htm#gene57) | 31 (0.54%) | ko00562 |
| 58 | [Carotenoid biosynthesis](../../../../F:%5C%E5%8D%8E%E5%A4%A7%E6%B5%8B%E5%BA%8F%5Ctranscriptome%20(trinity-Unigene-all)%5CCymbidium%5CCymbidium%5Cannotation%5CKEGG%5CCyTr-Unigene.fa.htm#gene58) | 31 (0.54%) | ko00906 |
| 59 | [Tryptophan metabolism](../../../../F:%5C%E5%8D%8E%E5%A4%A7%E6%B5%8B%E5%BA%8F%5Ctranscriptome%20(trinity-Unigene-all)%5CCymbidium%5CCymbidium%5Cannotation%5CKEGG%5CCyTr-Unigene.fa.htm#gene59) | 31 (0.54%) | ko00380 |
| 60 | [Citrate cycle (TCA cycle)](../../../../F:%5C%E5%8D%8E%E5%A4%A7%E6%B5%8B%E5%BA%8F%5Ctranscriptome%20(trinity-Unigene-all)%5CCymbidium%5CCymbidium%5Cannotation%5CKEGG%5CCyTr-Unigene.fa.htm#gene60) | 31 (0.54%) | ko00020 |
| 61 | [Ubiquinone and other terpenoid-quinone biosynthesis](../../../../F:%5C%E5%8D%8E%E5%A4%A7%E6%B5%8B%E5%BA%8F%5Ctranscriptome%20(trinity-Unigene-all)%5CCymbidium%5CCymbidium%5Cannotation%5CKEGG%5CCyTr-Unigene.fa.htm#gene61) | 31 (0.54%) | ko00130 |
| 62 | [Fatty acid metabolism](../../../../F:%5C%E5%8D%8E%E5%A4%A7%E6%B5%8B%E5%BA%8F%5Ctranscriptome%20(trinity-Unigene-all)%5CCymbidium%5CCymbidium%5Cannotation%5CKEGG%5CCyTr-Unigene.fa.htm#gene62) | 30 (0.52%) | ko00071 |
| 63 | [Proteasome](../../../../F:%5C%E5%8D%8E%E5%A4%A7%E6%B5%8B%E5%BA%8F%5Ctranscriptome%20(trinity-Unigene-all)%5CCymbidium%5CCymbidium%5Cannotation%5CKEGG%5CCyTr-Unigene.fa.htm#gene63) | 30 (0.52%) | ko03050 |
| 64 | [Selenoamino acid metabolism](../../../../F:%5C%E5%8D%8E%E5%A4%A7%E6%B5%8B%E5%BA%8F%5Ctranscriptome%20(trinity-Unigene-all)%5CCymbidium%5CCymbidium%5Cannotation%5CKEGG%5CCyTr-Unigene.fa.htm#gene64) | 28 (0.49%) | ko00450 |
| 65 | [N-Glycan biosynthesis](../../../../F:%5C%E5%8D%8E%E5%A4%A7%E6%B5%8B%E5%BA%8F%5Ctranscriptome%20(trinity-Unigene-all)%5CCymbidium%5CCymbidium%5Cannotation%5CKEGG%5CCyTr-Unigene.fa.htm#gene65) | 28 (0.49%) | ko00510 |
| 66 | [Valine, leucine and isoleucine biosynthesis](../../../../F:%5C%E5%8D%8E%E5%A4%A7%E6%B5%8B%E5%BA%8F%5Ctranscriptome%20(trinity-Unigene-all)%5CCymbidium%5CCymbidium%5Cannotation%5CKEGG%5CCyTr-Unigene.fa.htm#gene66) | 28 (0.49%) | ko00290 |
| 67 | [Propanoate metabolism](../../../../F:%5C%E5%8D%8E%E5%A4%A7%E6%B5%8B%E5%BA%8F%5Ctranscriptome%20(trinity-Unigene-all)%5CCymbidium%5CCymbidium%5Cannotation%5CKEGG%5CCyTr-Unigene.fa.htm#gene67) | 27 (0.47%) | ko00640 |
| 68 | [Valine, leucine and isoleucine degradation](../../../../F:%5C%E5%8D%8E%E5%A4%A7%E6%B5%8B%E5%BA%8F%5Ctranscriptome%20(trinity-Unigene-all)%5CCymbidium%5CCymbidium%5Cannotation%5CKEGG%5CCyTr-Unigene.fa.htm#gene68) | 27 (0.47%) | ko00280 |
| 69 | [SNARE interactions in vesicular transport](../../../../F:%5C%E5%8D%8E%E5%A4%A7%E6%B5%8B%E5%BA%8F%5Ctranscriptome%20(trinity-Unigene-all)%5CCymbidium%5CCymbidium%5Cannotation%5CKEGG%5CCyTr-Unigene.fa.htm#gene69) | 27 (0.47%) | ko04130 |
| 70 | [Pentose and glucuronate interconversions](../../../../F:%5C%E5%8D%8E%E5%A4%A7%E6%B5%8B%E5%BA%8F%5Ctranscriptome%20(trinity-Unigene-all)%5CCymbidium%5CCymbidium%5Cannotation%5CKEGG%5CCyTr-Unigene.fa.htm#gene70) | 27 (0.47%) | ko00040 |
| 71 | [Biosynthesis of unsaturated fatty acids](../../../../F:%5C%E5%8D%8E%E5%A4%A7%E6%B5%8B%E5%BA%8F%5Ctranscriptome%20(trinity-Unigene-all)%5CCymbidium%5CCymbidium%5Cannotation%5CKEGG%5CCyTr-Unigene.fa.htm#gene71) | 26 (0.45%) | ko01040 |
| 72 | [Regulation of autophagy](../../../../F:%5C%E5%8D%8E%E5%A4%A7%E6%B5%8B%E5%BA%8F%5Ctranscriptome%20(trinity-Unigene-all)%5CCymbidium%5CCymbidium%5Cannotation%5CKEGG%5CCyTr-Unigene.fa.htm#gene72) | 26 (0.45%) | ko04140 |
| 73 | [Natural killer cell mediated cytotoxicity](../../../../F:%5C%E5%8D%8E%E5%A4%A7%E6%B5%8B%E5%BA%8F%5Ctranscriptome%20(trinity-Unigene-all)%5CCymbidium%5CCymbidium%5Cannotation%5CKEGG%5CCyTr-Unigene.fa.htm#gene73) | 25 (0.44%) | ko04650 |
| 74 | [Fatty acid biosynthesis](../../../../F:%5C%E5%8D%8E%E5%A4%A7%E6%B5%8B%E5%BA%8F%5Ctranscriptome%20(trinity-Unigene-all)%5CCymbidium%5CCymbidium%5Cannotation%5CKEGG%5CCyTr-Unigene.fa.htm#gene74) | 25 (0.44%) | ko00061 |
| 75 | [Diterpenoid biosynthesis](../../../../F:%5C%E5%8D%8E%E5%A4%A7%E6%B5%8B%E5%BA%8F%5Ctranscriptome%20(trinity-Unigene-all)%5CCymbidium%5CCymbidium%5Cannotation%5CKEGG%5CCyTr-Unigene.fa.htm#gene75) | 24 (0.42%) | ko00904 |
| 76 | [Pantothenate and CoA biosynthesis](../../../../F:%5C%E5%8D%8E%E5%A4%A7%E6%B5%8B%E5%BA%8F%5Ctranscriptome%20(trinity-Unigene-all)%5CCymbidium%5CCymbidium%5Cannotation%5CKEGG%5CCyTr-Unigene.fa.htm#gene76) | 23 (0.4%) | ko00770 |
| 77 | [Tyrosine metabolism](../../../../F:%5C%E5%8D%8E%E5%A4%A7%E6%B5%8B%E5%BA%8F%5Ctranscriptome%20(trinity-Unigene-all)%5CCymbidium%5CCymbidium%5Cannotation%5CKEGG%5CCyTr-Unigene.fa.htm#gene77) | 21 (0.37%) | ko00350 |
| 78 | [Glyoxylate and dicarboxylate metabolism](../../../../F:%5C%E5%8D%8E%E5%A4%A7%E6%B5%8B%E5%BA%8F%5Ctranscriptome%20(trinity-Unigene-all)%5CCymbidium%5CCymbidium%5Cannotation%5CKEGG%5CCyTr-Unigene.fa.htm#gene78) | 20 (0.35%) | ko00630 |
| 79 | [Photosynthesis](../../../../F:%5C%E5%8D%8E%E5%A4%A7%E6%B5%8B%E5%BA%8F%5Ctranscriptome%20(trinity-Unigene-all)%5CCymbidium%5CCymbidium%5Cannotation%5CKEGG%5CCyTr-Unigene.fa.htm#gene79) | 20 (0.35%) | ko00195 |
| 80 | [Phenylalanine, tyrosine and tryptophan biosynthesis](../../../../F:%5C%E5%8D%8E%E5%A4%A7%E6%B5%8B%E5%BA%8F%5Ctranscriptome%20(trinity-Unigene-all)%5CCymbidium%5CCymbidium%5Cannotation%5CKEGG%5CCyTr-Unigene.fa.htm#gene80) | 20 (0.35%) | ko00400 |
| 81 | [Non-homologous end-joining](../../../../F:%5C%E5%8D%8E%E5%A4%A7%E6%B5%8B%E5%BA%8F%5Ctranscriptome%20(trinity-Unigene-all)%5CCymbidium%5CCymbidium%5Cannotation%5CKEGG%5CCyTr-Unigene.fa.htm#gene81) | 20 (0.35%) | ko03450 |
| 82 | [Steroid biosynthesis](../../../../F:%5C%E5%8D%8E%E5%A4%A7%E6%B5%8B%E5%BA%8F%5Ctranscriptome%20(trinity-Unigene-all)%5CCymbidium%5CCymbidium%5Cannotation%5CKEGG%5CCyTr-Unigene.fa.htm#gene82) | 20 (0.35%) | ko00100 |
| 83 | [beta-Alanine metabolism](../../../../F:%5C%E5%8D%8E%E5%A4%A7%E6%B5%8B%E5%BA%8F%5Ctranscriptome%20(trinity-Unigene-all)%5CCymbidium%5CCymbidium%5Cannotation%5CKEGG%5CCyTr-Unigene.fa.htm#gene83) | 20 (0.35%) | ko00410 |
| 84 | [Sphingolipid metabolism](../../../../F:%5C%E5%8D%8E%E5%A4%A7%E6%B5%8B%E5%BA%8F%5Ctranscriptome%20(trinity-Unigene-all)%5CCymbidium%5CCymbidium%5Cannotation%5CKEGG%5CCyTr-Unigene.fa.htm#gene84) | 19 (0.33%) | ko00600 |
| 85 | [Terpenoid backbone biosynthesis](../../../../F:%5C%E5%8D%8E%E5%A4%A7%E6%B5%8B%E5%BA%8F%5Ctranscriptome%20(trinity-Unigene-all)%5CCymbidium%5CCymbidium%5Cannotation%5CKEGG%5CCyTr-Unigene.fa.htm#gene85) | 18 (0.31%) | ko00900 |
| 86 | [Other glycan degradation](../../../../F:%5C%E5%8D%8E%E5%A4%A7%E6%B5%8B%E5%BA%8F%5Ctranscriptome%20(trinity-Unigene-all)%5CCymbidium%5CCymbidium%5Cannotation%5CKEGG%5CCyTr-Unigene.fa.htm#gene86) | 18 (0.31%) | ko00511 |
| 87 | [Glucosinolate biosynthesis](../../../../F:%5C%E5%8D%8E%E5%A4%A7%E6%B5%8B%E5%BA%8F%5Ctranscriptome%20(trinity-Unigene-all)%5CCymbidium%5CCymbidium%5Cannotation%5CKEGG%5CCyTr-Unigene.fa.htm#gene87) | 17 (0.3%) | ko00966 |
| 88 | [Sulfur metabolism](../../../../F:%5C%E5%8D%8E%E5%A4%A7%E6%B5%8B%E5%BA%8F%5Ctranscriptome%20(trinity-Unigene-all)%5CCymbidium%5CCymbidium%5Cannotation%5CKEGG%5CCyTr-Unigene.fa.htm#gene88) | 16 (0.28%) | ko00920 |
| 89 | [Glycosaminoglycan degradation](../../../../F:%5C%E5%8D%8E%E5%A4%A7%E6%B5%8B%E5%BA%8F%5Ctranscriptome%20(trinity-Unigene-all)%5CCymbidium%5CCymbidium%5Cannotation%5CKEGG%5CCyTr-Unigene.fa.htm#gene89) | 15 (0.26%) | ko00531 |
| 90 | [Glycosylphosphatidylinositol(GPI)-anchor biosynthesis](../../../../F:%5C%E5%8D%8E%E5%A4%A7%E6%B5%8B%E5%BA%8F%5Ctranscriptome%20(trinity-Unigene-all)%5CCymbidium%5CCymbidium%5Cannotation%5CKEGG%5CCyTr-Unigene.fa.htm#gene90) | 14 (0.24%) | ko00563 |
| 91 | [Flavone and flavonol biosynthesis](../../../../F:%5C%E5%8D%8E%E5%A4%A7%E6%B5%8B%E5%BA%8F%5Ctranscriptome%20(trinity-Unigene-all)%5CCymbidium%5CCymbidium%5Cannotation%5CKEGG%5CCyTr-Unigene.fa.htm#gene91) | 13 (0.23%) | ko00944 |
| 92 | [Tropane, piperidine and pyridine alkaloid biosynthesis](../../../../F:%5C%E5%8D%8E%E5%A4%A7%E6%B5%8B%E5%BA%8F%5Ctranscriptome%20(trinity-Unigene-all)%5CCymbidium%5CCymbidium%5Cannotation%5CKEGG%5CCyTr-Unigene.fa.htm#gene92) | 12 (0.21%) | ko00960 |
| 93 | [Nicotinate and nicotinamide metabolism](../../../../F:%5C%E5%8D%8E%E5%A4%A7%E6%B5%8B%E5%BA%8F%5Ctranscriptome%20(trinity-Unigene-all)%5CCymbidium%5CCymbidium%5Cannotation%5CKEGG%5CCyTr-Unigene.fa.htm#gene93) | 12 (0.21%) | ko00760 |
| 94 | [Anthocyanin biosynthesis](../../../../F:%5C%E5%8D%8E%E5%A4%A7%E6%B5%8B%E5%BA%8F%5Ctranscriptome%20(trinity-Unigene-all)%5CCymbidium%5CCymbidium%5Cannotation%5CKEGG%5CCyTr-Unigene.fa.htm#gene94) | 11 (0.19%) | ko00942 |
| 95 | [Histidine metabolism](../../../../F:%5C%E5%8D%8E%E5%A4%A7%E6%B5%8B%E5%BA%8F%5Ctranscriptome%20(trinity-Unigene-all)%5CCymbidium%5CCymbidium%5Cannotation%5CKEGG%5CCyTr-Unigene.fa.htm#gene95) | 11 (0.19%) | ko00340 |
| 96 | [Riboflavin metabolism](../../../../F:%5C%E5%8D%8E%E5%A4%A7%E6%B5%8B%E5%BA%8F%5Ctranscriptome%20(trinity-Unigene-all)%5CCymbidium%5CCymbidium%5Cannotation%5CKEGG%5CCyTr-Unigene.fa.htm#gene96) | 10 (0.17%) | ko00740 |
| 97 | [One carbon pool by folate](../../../../F:%5C%E5%8D%8E%E5%A4%A7%E6%B5%8B%E5%BA%8F%5Ctranscriptome%20(trinity-Unigene-all)%5CCymbidium%5CCymbidium%5Cannotation%5CKEGG%5CCyTr-Unigene.fa.htm#gene97) | 10 (0.17%) | ko00670 |
| 98 | [Arachidonic acid metabolism](../../../../F:%5C%E5%8D%8E%E5%A4%A7%E6%B5%8B%E5%BA%8F%5Ctranscriptome%20(trinity-Unigene-all)%5CCymbidium%5CCymbidium%5Cannotation%5CKEGG%5CCyTr-Unigene.fa.htm#gene98) | 9 (0.16%) | ko00590 |
| 99 | [Benzoxazinoid biosynthesis](../../../../F:%5C%E5%8D%8E%E5%A4%A7%E6%B5%8B%E5%BA%8F%5Ctranscriptome%20(trinity-Unigene-all)%5CCymbidium%5CCymbidium%5Cannotation%5CKEGG%5CCyTr-Unigene.fa.htm#gene99) | 9 (0.16%) | ko00402 |
| 100 | [Thiamine metabolism](../../../../F:%5C%E5%8D%8E%E5%A4%A7%E6%B5%8B%E5%BA%8F%5Ctranscriptome%20(trinity-Unigene-all)%5CCymbidium%5CCymbidium%5Cannotation%5CKEGG%5CCyTr-Unigene.fa.htm#gene100) | 9 (0.16%) | ko00730 |
| 101 | [Indole alkaloid biosynthesis](../../../../F:%5C%E5%8D%8E%E5%A4%A7%E6%B5%8B%E5%BA%8F%5Ctranscriptome%20(trinity-Unigene-all)%5CCymbidium%5CCymbidium%5Cannotation%5CKEGG%5CCyTr-Unigene.fa.htm#gene101) | 9 (0.16%) | ko00901 |
| 102 | [Isoquinoline alkaloid biosynthesis](../../../../F:%5C%E5%8D%8E%E5%A4%A7%E6%B5%8B%E5%BA%8F%5Ctranscriptome%20(trinity-Unigene-all)%5CCymbidium%5CCymbidium%5Cannotation%5CKEGG%5CCyTr-Unigene.fa.htm#gene102) | 9 (0.16%) | ko00950 |
| 103 | [Glycosphingolipid biosynthesis - ganglio series](../../../../F:%5C%E5%8D%8E%E5%A4%A7%E6%B5%8B%E5%BA%8F%5Ctranscriptome%20(trinity-Unigene-all)%5CCymbidium%5CCymbidium%5Cannotation%5CKEGG%5CCyTr-Unigene.fa.htm#gene103) | 8 (0.14%) | ko00604 |
| 104 | [Folate biosynthesis](../../../../F:%5C%E5%8D%8E%E5%A4%A7%E6%B5%8B%E5%BA%8F%5Ctranscriptome%20(trinity-Unigene-all)%5CCymbidium%5CCymbidium%5Cannotation%5CKEGG%5CCyTr-Unigene.fa.htm#gene104) | 8 (0.14%) | ko00790 |
| 105 | [Lysine biosynthesis](../../../../F:%5C%E5%8D%8E%E5%A4%A7%E6%B5%8B%E5%BA%8F%5Ctranscriptome%20(trinity-Unigene-all)%5CCymbidium%5CCymbidium%5Cannotation%5CKEGG%5CCyTr-Unigene.fa.htm#gene105) | 8 (0.14%) | ko00300 |
| 106 | [Photosynthesis - antenna proteins](../../../../F:%5C%E5%8D%8E%E5%A4%A7%E6%B5%8B%E5%BA%8F%5Ctranscriptome%20(trinity-Unigene-all)%5CCymbidium%5CCymbidium%5Cannotation%5CKEGG%5CCyTr-Unigene.fa.htm#gene106) | 6 (0.1%) | ko00196 |
| 107 | [Brassinosteroid biosynthesis](../../../../F:%5C%E5%8D%8E%E5%A4%A7%E6%B5%8B%E5%BA%8F%5Ctranscriptome%20(trinity-Unigene-all)%5CCymbidium%5CCymbidium%5Cannotation%5CKEGG%5CCyTr-Unigene.fa.htm#gene107) | 6 (0.1%) | ko00905 |
| 108 | [Monoterpenoid biosynthesis](../../../../F:%5C%E5%8D%8E%E5%A4%A7%E6%B5%8B%E5%BA%8F%5Ctranscriptome%20(trinity-Unigene-all)%5CCymbidium%5CCymbidium%5Cannotation%5CKEGG%5CCyTr-Unigene.fa.htm#gene108) | 4 (0.07%) | ko00902 |
| 109 | [Synthesis and degradation of ketone bodies](../../../../F:%5C%E5%8D%8E%E5%A4%A7%E6%B5%8B%E5%BA%8F%5Ctranscriptome%20(trinity-Unigene-all)%5CCymbidium%5CCymbidium%5Cannotation%5CKEGG%5CCyTr-Unigene.fa.htm#gene109) | 4 (0.07%) | ko00072 |
| 110 | [Polyketide sugar unit biosynthesis](../../../../F:%5C%E5%8D%8E%E5%A4%A7%E6%B5%8B%E5%BA%8F%5Ctranscriptome%20(trinity-Unigene-all)%5CCymbidium%5CCymbidium%5Cannotation%5CKEGG%5CCyTr-Unigene.fa.htm#gene110) | 4 (0.07%) | ko00523 |
| 111 | [Vitamin B6 metabolism](../../../../F:%5C%E5%8D%8E%E5%A4%A7%E6%B5%8B%E5%BA%8F%5Ctranscriptome%20(trinity-Unigene-all)%5CCymbidium%5CCymbidium%5Cannotation%5CKEGG%5CCyTr-Unigene.fa.htm#gene111) | 4 (0.07%) | ko00750 |
| 112 | [C5-Branched dibasic acid metabolism](../../../../F:%5C%E5%8D%8E%E5%A4%A7%E6%B5%8B%E5%BA%8F%5Ctranscriptome%20(trinity-Unigene-all)%5CCymbidium%5CCymbidium%5Cannotation%5CKEGG%5CCyTr-Unigene.fa.htm#gene112) | 4 (0.07%) | ko00660 |
| 113 | [Betalain biosynthesis](../../../../F:%5C%E5%8D%8E%E5%A4%A7%E6%B5%8B%E5%BA%8F%5Ctranscriptome%20(trinity-Unigene-all)%5CCymbidium%5CCymbidium%5Cannotation%5CKEGG%5CCyTr-Unigene.fa.htm#gene113) | 3 (0.05%) | ko00965 |
| 114 | [Fatty acid elongation in mitochondria](../../../../F:%5C%E5%8D%8E%E5%A4%A7%E6%B5%8B%E5%BA%8F%5Ctranscriptome%20(trinity-Unigene-all)%5CCymbidium%5CCymbidium%5Cannotation%5CKEGG%5CCyTr-Unigene.fa.htm#gene114) | 3 (0.05%) | ko00062 |
| 115 | [Biotin metabolism](../../../../F:%5C%E5%8D%8E%E5%A4%A7%E6%B5%8B%E5%BA%8F%5Ctranscriptome%20(trinity-Unigene-all)%5CCymbidium%5CCymbidium%5Cannotation%5CKEGG%5CCyTr-Unigene.fa.htm#gene115) | 3 (0.05%) | ko00780 |
| 116 | [Caffeine metabolism](../../../../F:%5C%E5%8D%8E%E5%A4%A7%E6%B5%8B%E5%BA%8F%5Ctranscriptome%20(trinity-Unigene-all)%5CCymbidium%5CCymbidium%5Cannotation%5CKEGG%5CCyTr-Unigene.fa.htm#gene116) | 3 (0.05%) | ko00232 |
| 117 | [Lipoic acid metabolism](../../../../F:%5C%E5%8D%8E%E5%A4%A7%E6%B5%8B%E5%BA%8F%5Ctranscriptome%20(trinity-Unigene-all)%5CCymbidium%5CCymbidium%5Cannotation%5CKEGG%5CCyTr-Unigene.fa.htm#gene117) | 2 (0.03%) | ko00785 |
| 118 | [Taurine and hypotaurine metabolism](../../../../F:%5C%E5%8D%8E%E5%A4%A7%E6%B5%8B%E5%BA%8F%5Ctranscriptome%20(trinity-Unigene-all)%5CCymbidium%5CCymbidium%5Cannotation%5CKEGG%5CCyTr-Unigene.fa.htm#gene118) | 2 (0.03%) | ko00430 |
| 119 | [Glycosphingolipid biosynthesis - globo series](../../../../F:%5C%E5%8D%8E%E5%A4%A7%E6%B5%8B%E5%BA%8F%5Ctranscriptome%20(trinity-Unigene-all)%5CCymbidium%5CCymbidium%5Cannotation%5CKEGG%5CCyTr-Unigene.fa.htm#gene119) | 1 (0.02%) | ko00603 |
