# Supplementary material for: Deep sequencing–based comparative transcriptional profiles of Cymbidium hybridum roots in response to mycorrhizal and non-mycorrhizal beneficial fungi
Source: BMC Genomics. 2014 Aug 31;15(1):747. doi: 10.1186/1471-2164-15-747 (PMC4162972; doi:10.1186/1471-2164-15-747)
Supplement: Supplementary file 16 — Additional file 16: Figure S7-S9: Scatterplot for top 20 enriched KEGG pathways of DEGs in the interactions between C. hybridum and ML01 (S7), ZH3A-3 (S8) or ML01 + ZH3A-3 (S9). (ZIP 22 KB) [file 12864_2014_6428_MOESM16_ESM.zip › Additional file 16/Figure S8 CyEX22vsCyEX20.DEG_enriched_KEGG_pathway_scatterplot.pdf]

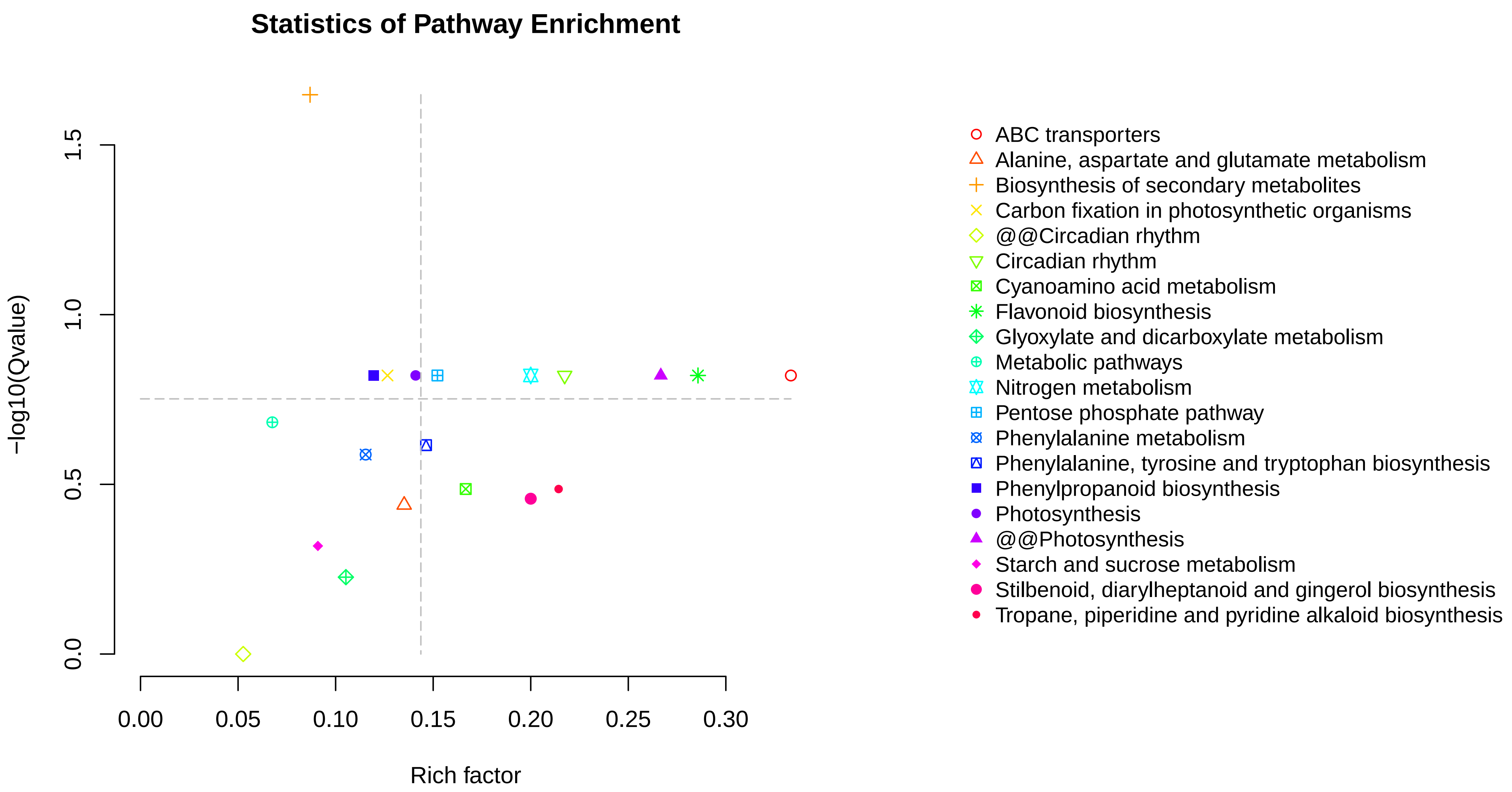
Statistics of Pathway Enrichment
−log10(Qvalue)
0.0
0.5
1.0
1.5
0.00
0.05
0.10
0.15
0.20
0.25
0.30
Rich factor
ABC transporters
Alanine, aspartate and glutamate metabolism
Biosynthesis of secondary metabolites
Carbon fixation in photosynthetic organisms
@@Circadian rhythm
Circadian rhythm
Cyanoamino acid metabolism
Flavonoid biosynthesis
Glyoxylate and dicarboxylate metabolism
Metabolic pathways
Nitrogen metabolism
Pentose phosphate pathway
Phenylalanine metabolism
Phenylalanine, tyrosine and tryptophan biosynthesis
Phenylpropanoid biosynthesis
Photosynthesis
@@Photosynthesis
Starch and sucrose metabolism
Stilbenoid, diarylheptanoid and gingerol biosynthesis
Tropane, piperidine and pyridine alkaloid biosynthesis
