# Supplementary material for: Deep sequencing–based comparative transcriptional profiles of Cymbidium hybridum roots in response to mycorrhizal and non-mycorrhizal beneficial fungi
Source: BMC Genomics. 2014 Aug 31;15(1):747. doi: 10.1186/1471-2164-15-747 (PMC4162972; doi:10.1186/1471-2164-15-747)
Supplement: Supplementary file 19 — Additional file 19: Figure S11: Phylogenetic tree for the amino acid sequences of symbiosis- associated phosphate transporters from plant and fungi.The dendrogram was generated by Mega 4.0 software using ClustlW for the alignment and the neighbor-joining method for the construction of phylogeny. The plant Pi-transporters accession numbers could be obtained in [49]. Fungal phosphate transporters accession numbers: Glomus versiforme: GvePT, AAC49132.1; GvePT2, gi|74654973; Rhizophagus intraradices: RinPT, AAL37552.1; Yeast: YEPHO84, gi|1346710; Sesbania rostrata: SerPT, CAC28219.1; Piriformospora indica: PiPT1, ABI93950.1; PiPT2, CCA74169.1; PiPT3, CCA74178.1; PiPT4, CCA76379.1. The nucleotide sequences of putative phosphate transporters in our study were available in Additional file 6: Table S3. (PDF 90 KB) [file 12864_2014_6428_MOESM19_ESM.pdf]

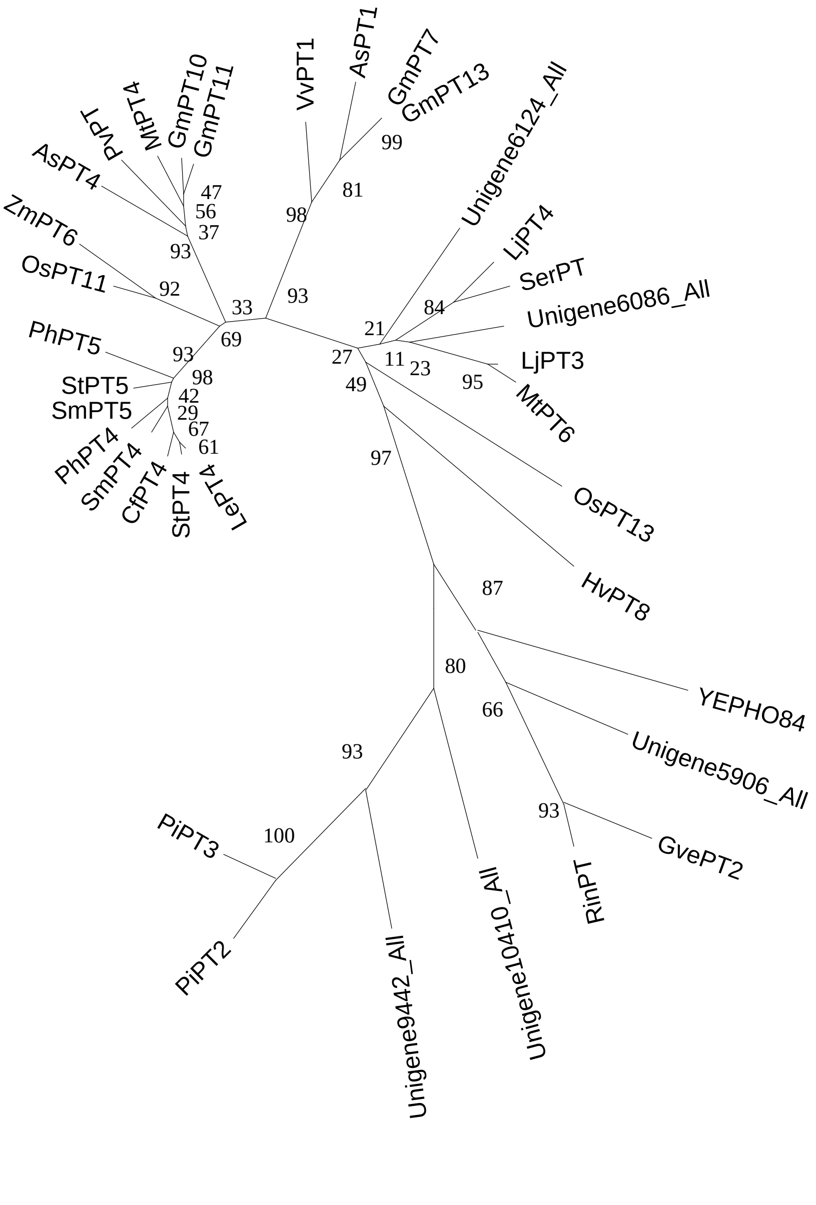
VvPT1
AsPT1
GmPT7
GmPT13
Unigene6124_All
LjPT4
SerPT
Unigene6086_All
LjPT3
MtPT6
OsPT13
HvPT8
YEPHO84
Unigene5906_All
GvePT2
RinPT
Unigene10410_All
Unigene9442_All
PiPT2
PiPT3
LePT4
StPT4
CfPT4
SmPT4
PhPT4
SmPT5
StPT5
PhPT5
OsPT11
ZmPT6
AsPT4
PvPT
MtPT4
GmPT10
GmPT11
0.1
